# Flat Band Generation Through Interlayer Geometric Frustration in Intercalated Transition Metal Dichalcogenides

**DOI:** 10.1002/smll.202409535

**Published:** 2025-01-26

**Authors:** Yawen Peng, Ren He, Peng Li, Sergey Zhdanovich, Matteo Michiardi, Sergey Gorovikov, Marta Zonno, Andrea Damascelli, Guo‐Xing Miao

**Affiliations:** ^1^ Institute for Quantum Computing and Department of Electrical and Computer Engineering University of Waterloo Waterloo ON N2L3G1 Canada; ^2^ Quantum Matter Institute University of British Columbia Vancouver BC V6T 1Z4 Canada; ^3^ Canadian Light Source Inc 44 Innovation Boulevard Saskatoon SK S7N 2V3 Canada

**Keywords:** ARPES, DFT calculations, flat‐bands, tight‐binding model, transition metal dichalcogenide

## Abstract

Electronic flat bands can lead to rich many‐body quantum phases by quenching the electron's kinetic energy and enhancing many‐body correlation. The reduced bandwidth can be realized by either destructive quantum interference in frustrated lattices, or by generating heavy band folding with avoided band crossing in Moiré superlattices. Here a general approach is proposed to introduce flat bands into widely studied transition metal dichalcogenide (TMD) materials by dilute intercalation. A flat band with vanishing dispersion is observed by angle‐resolved photoemission spectroscopy (ARPES) over the entire momentum space in intercalated Mn_1/4_TaS_2_. Polarization‐dependent ARPES measurements combined with symmetry analysis reveal the orbital characters of the flat band. Supercell tight‐binding simulations suggest that such flat bands arising from destructive interference between Mn and Ta on S through hopping pathways, are ubiquitous in a range of TMD families as well as for different intercalation configurations. The findings establish a new material platform to manipulate flat band structures and explore their corresponding emergent correlated properties.

## Graphical abstract

With a tight binding approach, a theoretical framework is laid down for the universal existence of flat bands in intercalated transition metal dichalcogenides. The transition metal *d*‐orbitals form destructive interference via the chalcogenide *p*‐orbitals, typically over an extended triangular lattice depending on the nature of intercalation. This concept is experimentally verified in a model system of 2x2 Mn intercalated TaS2.

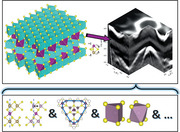

## 1 Introduction

Quantum many‐body physics with strong electron correlation gives rise to a wide range of exotic electronic properties such as unconventional superconductivity and magnetism.^[^ ^1^, ^2^, ^3^, ^4^, ^5^ ^]^ This can be achieved in flat band materials characterized by bands with vanishing energy dispersion in momentum space and located near the Femi level. The kinetic energy of electrons is strongly suppressed due to extremely heavy effective mass and gives way to enhanced correlation effects.

The flat band phenomenology has gained significant attention after its experimental realization and observation recently. Except for the heavy fermion compounds with 4f electrons, there are generally two types of 2D materials that host flat bands. One is a material with a unique frustrated lattice such as the widely studied kagome metal,^[^ ^6^, ^7^, ^8^, ^9^, ^10^, ^11^ ^]^ and the other one is a stacked Moiré superlattice such as twisted bilayer graphene (TBG).^[^ ^12^, ^13^, ^14^, ^15^ ^]^ The 2D kagome lattice itself has intrinsic topological flat bands due to structural geometric frustration and lattice topology. The ability to tune flat band to the Femi level is limited because of its intrinsic property. While the TBG has an extrinsic flat band introduced by modifying interlayer interactions and Moiré pattern engineering, with small perturbation to the original Dirac Fermion nature of graphene. By applying electrostatic gating, the Fermi level can be shifted and different filling factors of flat band can be achieved.

Here we report a novel material platform that shows the emergence of flat bands in TMD compounds upon intercalation, different from above two systems. By combining angle‐resolved photoemission spectroscopy (ARPES) with density functional theory (DFT) and tight binding calculations, we study the model system of 2 × 2 Mn_1/4_TaS_2_ and establish the ubiquitous existence of flat bands in intercalated TMDs. The origin and property of the flat band have similarities with that in kagome metal and Moiré superlattice: while the formation is caused by an intrinsic crystal structural factor that the symmetrically aligned Mn and Ta can have destructive wavefunction cancellation on S, the flat band introduced by Mn intercalation has negligible modification to the original band structure of host material TaS_2_. And the ability of tuning flat band to the Fermi level in such intercalated TMDs can be achieved by varying the intercalant/TMD species and intercalation concentration.

Our ARPES results show a flat band located 1.23 eV below the Fermi level, whose orbital characters experimentally determined via polarization‐dependent ARPES measurements well agree with orbital projected DFT calculations as well as crystal symmetry analysis. Furthermore, our tight‐binding modeling reveals that these flat bands can be generalized to other TMD families and intercalation cases, including H or T phase TMDs, H_a_ or H_c_ interlayer stacking sequence, and $\sqrt{3}\;$×$\;\sqrt{3}$ or any other supercell reconstructions. These findings establish a generic way to introduce and manipulate flat‐band electronic structures in intercalated TMDs, and shed important light on exploring unique correlated phenomena in these materials.

### 1.1 Flat bands in Mn_1/4_TaS_2_

**Figure 1:**
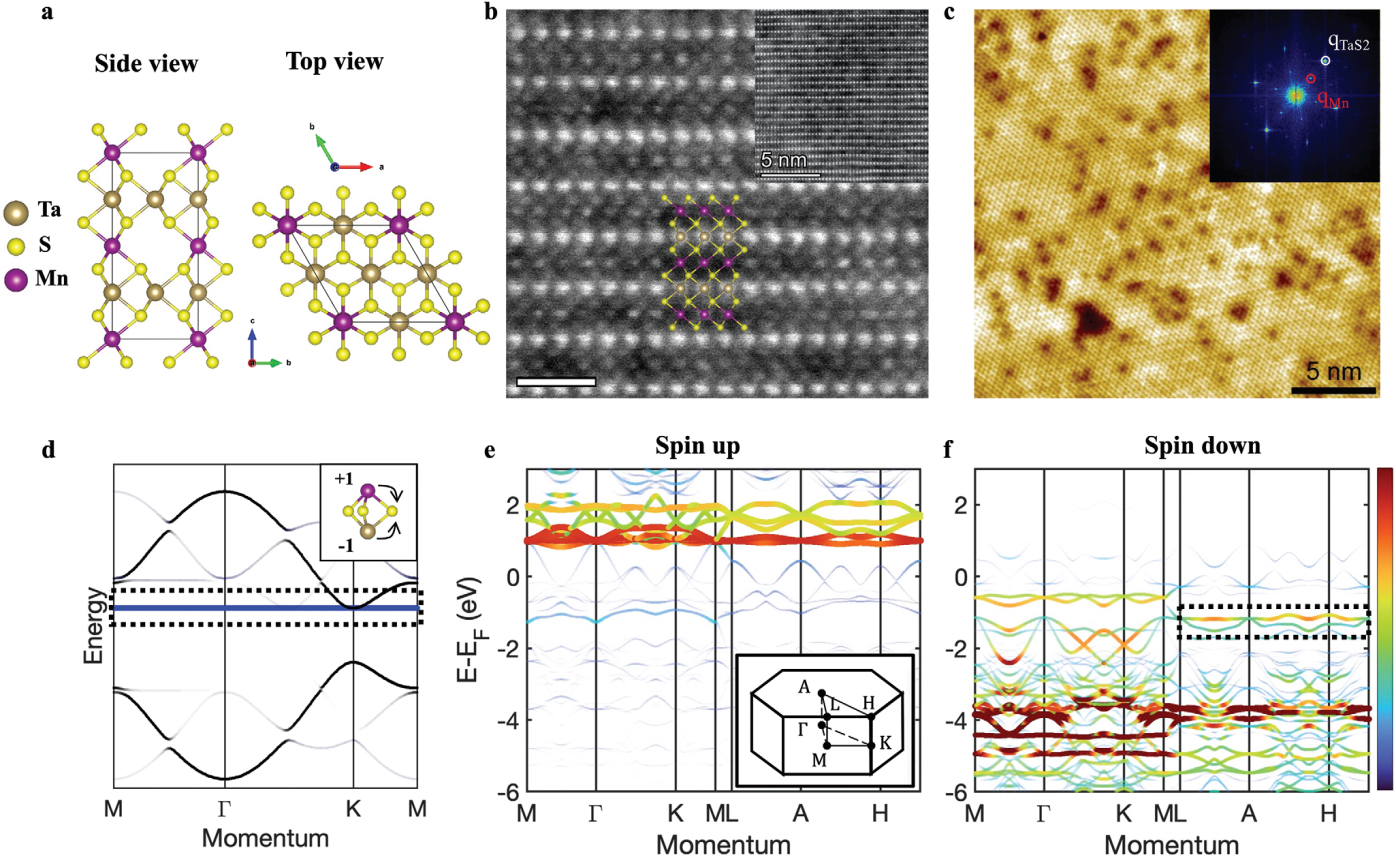
Crystal characterization and band structure calculation. a) Schematic of the crystal structure of Mn_1/4_TaS_2_. b) STEM cross‐section view of Mn_1/4_TaS_2_ along [100] axis. The crystal structure is overlaid with the image. The scale bar is 1nm. The inset is the large‐scale STEM image with a scale bar 5 nm. c) STM atomic resolution of TaS_2_ termination (V_bias_ = 1 V, I_set_ = 0.5 A). The scale bar is 5nm. The inset is the corresponding FFT pattern. White and red circles denote 1 × 1 TaS_2_ and 2 × 2 Mn lattice. d) Unfolded band structure of the 9‐band tight‐binding model (same parameters used in Figure S2, Supporting Information). The flat band with a blue color is indicated by the dashed box. The inset shows the localization due to destructive interference on S. The amplitudes are not necessary to be equal, depending on Mn‐S/Ta‐S hopping difference. e,f) DFT band structures of Mn_1/4_TaS_2_ with (e) spin up and (f) spin down components. The line width and color represent flat band weight projections of Mn. Inset in (e) is the 3D Brillion zone. The observed flat band in ARPES is indicated by the dashed box in f.

The crystal structure of Mn_1/4_TaS_2_ (**Figure** **1**a) contains the host compound 2H TaS_2_ as well as Mn atoms intercalated into the van der Waals (vdW) gaps, which form an ordered 2 × 2 periodic sublattice. The successful intercalation is confirmed by X‐ray diffraction (XRD, see Figure S1, Supporting Information) and high‐resolution cross‐section scanning transmission electron microscopy (STEM) measurement (Figure 1b), with intercalated Mn aligned with Ta in the *c*‐direction^[^ ^16^ ^]^ Scanning tunneling microscopy (STM) measurements on the (001) cleaved surface show a clear 2 × 2 periodicity of the Mn layer under the TaS2‐terminated surface (Figure 1c). The measured lattice constant of 2 × 2 Mn (Figure S1, Supporting Information) is 6.6 Å which is twice that of TaS_2_.

To study the electronic structure of Mn_1/4_TaS_2_, we start by performing the DFT calculation and tight binding simulation. The DFT calculated band structure without spin polarization mainly consists of host TaS_2_ dispersion with additional folded bands due to the larger supercell. Several flat bands are observed near the Fermi level which are mainly from Mn 3d orbitals (Figure S1, Supporting Information). To understand the origin of the flat band, a 9‐band tight‐binding Hamiltonian with only *s*‐orbitals and nearest neighbor hopping is constructed (see Note SI, Supporting Information). The toy model contains 4 Ta, 4 S, and 1 Mn atoms, forming a honeycomb‐like Ta‐S layer with 2 × 2 Mn stacked on top of Ta and connected via S (Figure S2, Supporting Information), which is the building block of the unit cell. This *s*‐orbital tight binding model can be generalized to *d*‐orbital one, because each Mn has a Ta symmetrically aligned with respect to the S plane, therefore sharing same hopping phases regardless *s* or *d* orbitals.

The tight binding band structure is given in Figure 1d. Two dispersive bands (black‐gray curves) come from the original honeycomb‐like Ta‐S lattice with a gap opening at K point, while the flat band (blue curve) originates from the destructive interference between Mn and Ta orbitals (Figure 1d inset). The dilute intercalation ensures no interaction between Mn–Mn lattices (set to zero in this toy model), while the hopping between Mn‐S is destructively canceled by that of Ta‐S directly below. This makes wave function localized in the Mn‐S‐Ta trigonal bipyramidal structure and cannot propagate beyond S edges.

The flat band is strongly related to the onsite potential of Mn (ɛ_*Mn*_) and will move accordingly when ɛ_*Mn*_ Changes (see Note SI and Figure S3, Supporting Information). The simplified tight‐binding model is consistent with the spin‐polarized DFT calculation (Figure 1e,f). The flat bands mostly have contributions from Mn *d*‐orbitals. Due to the exchange splitting of Mn 3d electrons, the flat bands split into two groups with opposite spin polarizations. The spin‐up component has higher energy which is closer to the onsite potential of the Ta 5d electrons according to Wannier calculations, so this set of flat bands moves upward above the Fermi level and becomes flatter. On the contrary, the spin‐down component has lower energy and a larger energy difference with the Ta 5d orbitals, so these flat bands move downward and become more dispersive, affected by hybridization with the S bands as well.

**Figure 2:**
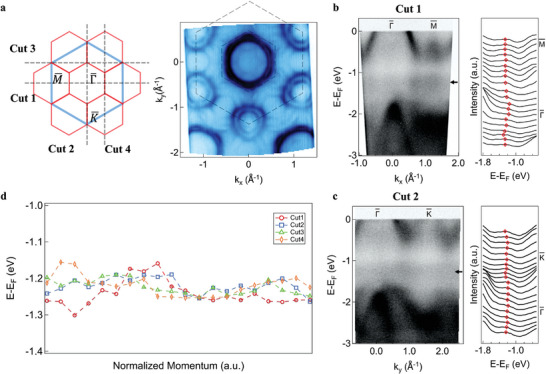
Flat bands in Mn_1/4_TaS_2_ revealed by ARPES. a) (Left) Schematic of 2D Brillouin zone of Mn_1/4_TaS_2_ with high‐symmetry points labeled. The blue hexagon represents the primitive TaS_2_ Brillouin zone and the blue hexagons represent the reduced ones of intercalated Mn_1/4_TaS_2_. Dashed lines indicate ARPES momentum‐space cuts directions in (b,c), as well as in Figure S4 (Supporting Information). (Right) Fermi surface of Mn_1/4_TaS_2_. Dashed lines indicate the primitive and reduced Brillouin zones. b,c) Different ARPES spectra (Cut 1 and 2) with corresponding EDCs along high‐symmetry directions. Black arrows mark the flat band energy positions, and red rhombus dots track the flat band peaks in EDCs. d) Evolutions of the peak positions in Cut 1–4. The black solid line indicates the approximate position of the flat band. All the data were acquired with linear horizontal photons with an energy of 75 eV.

The existence and the origin of flat bands in Mn_1/4_TaS_2_ revealed via DFT and tight‐binding simulations are further confirmed by ARPES measurements presented in **Figure** **2**. Due to the supercell folding, the pristine Brillouin zone is reduced into a smaller hexagon as shown in Figure 2a. The two hexagonal barrels centered at the Gamma point and the two ring‐shaped barrels centered at the K points are the typical features of the host 2H TaS_2_ with spin‐orbital coupling splitting. However, looking closely, there exist additional weaker arcs caused by the folding of these original TaS_2_ bands. The barrels around the K points are folded around the Gamma point and form a new hexagonal electron pocket. In addition, the Gamma point barrels are folded to M points, forming several arcs that connect barrels at different K points on the Fermi surface.

Different cuts across the Brillouin zone are examined and a flat band is identified over the whole momentum space. Figure 2b,c give the ARPES spectra measured along high‐symmetry paths $\bar{\Gamma }-\bar{M}$ and $\bar{\Gamma }-\bar{K}$ respectively. The flat bands indicated by black arrows fall right inside the TaS_2_ gap formed between bands crossing the Fermi level with primarily Ta d_z2_ nature (‐0.7 eV and above) and those with primarily S p orbitals natures (‐1.5 eV and below). To better visualize the dispersionless band structures, the stackings of energy distribution curves (EDCs) for different cuts are presented (Figure 2b,c). The flat bands manifest as peaks whose positions are tracked by Lorentzian fittings (see ARPES EDC peak fitting in Experimental Section and Table. S1, Supporting Information) of the integrated EDCs. Other spectra taken across high‐symmetry points (Cut 3 and 4 along the directions shown in Figure 2a) are examined in Figure S4 (Supporting Information). The second derivative plots of all these cuts are provided as well (Figure S5, Supporting Information), where the flat bands have improved visibility and get better resolved. The evolutions of flat band peak positions from the fittings are summarized in Figure 2d. The flat bands are located ≈1.23 eV below the Fermi level, and they exhibit a negligible dispersion in energy throughout the whole momentum space, again confirming the flat band nature in the Mn_1/4_TaS_2_. The bandwidth of the flat band is estimated to be 0.15 eV. Broadening can be attributed to the presence of the next nearest neighbor (NNN) hopping (see Note SII and Figure S3, Supporting Information) and a k_z_ dispersion due to interlayer hopping (see Note SV, Figures S12, S13, Supporting Information). Overall, the ARPES measurements provide experimental evidence of flat bands in this intercalated TMD system, and are consistent with both the theoretical DFT calculation and the simplified tight‐binding modeling.

### 1.2 Polarization‐Dependent ARPES Measurement

**Figure 3:**
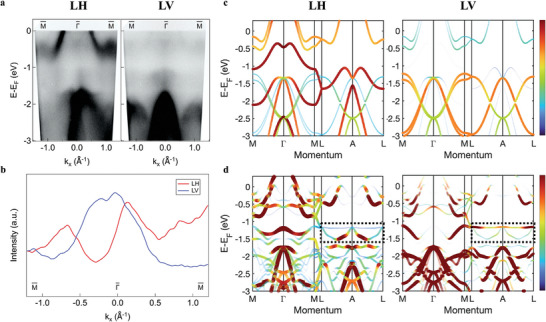
Polarization dependence and orbital characters of flat bands. a) ARPES spectrums measured along $\bar{M}-\bar{\Gamma }-\bar{M}$ direction with (left) LH and (right) LV polarization. b), MDCs at the flat band position (integrated over an energy range of 40 meV ≈‐1.23 eV) in (red) LH and (blue) LV polarizations. **c**) DFT band structures of TaS_2_ along M‐Γ‐M/L‐A‐L directions with LH and LV polarizations respectively. Line width and color represent even/odd orbital weight projections in LH/LV polarization. d) Projected DFT band structures of 2 × 2 Mn_1/4_TaS_2_ with band unfolding in LH/LV polarizations. Dashed boxes indicate the flat band positions.

In addition to the existence of flat bands, we noticed that there are some variations of their spectral intensities across momentum space. To better understand the origins and properties of the flat bands, the ARPES band dispersion measurements along the $\bar{M}-\bar{\Gamma }-\bar{M}$ and $\bar{K^{\prime }}-\bar{K}-\bar{K^{\prime }}$ (which is parallel to $\bar{M}-\bar{\Gamma }-\bar{M}$) directions are performed with both linear vertical and horizontal polarizations of the incident light. In the linear horizontal (LH) polarization, the flat band is suppressed in intensity near the Gamma point (**Figure** **3**a). As a comparison, when the incident photon is linear vertical (LV) polarized, the spectral intensity near the Gamma point becomes enhanced. This feature gets resolved more clearly in the momentum distribution curve (MDC) at the flat band positions. Figure 3b gives the comparison of MDCs extracted from both ARPES spectra: the dip near the Gamma point observed with LH polarization (red curve) transforms into a hump in LV polarization (blue). The intensity asymmetry in LH polarization is due to the matrix element effect, that the photoemission intensity depends on the angle between the momentum of the electron and the electric field vector of the photon. The same behavior of spectra intensity variation is found at K point as well (Figure S6, Supporting Information). The overall flat band intensities compensate for each other and fill up the whole momentum space (see Figure S7, Supporting Information).

This intensity variation is caused by the dipole selection rules in linearly polarized photoemission and can be used to reveal the orbital characters of the flat bands. In the experimental set‐up used for our polarization‐dependent ARPES measurements (Figure S8, Supporting Information), the LH (LV) polarization has even (odd) parity with respect to the mirror plane (the beam incident *xz* plane in the illustration), which means only those bands that have the same even (odd) parity can be selectively resolved. Then the *d*‐and *p* orbitals can be divided according to their parity into two groups which are d_z2_, d_x2‐y2_, d_xz_, p_z_, p_x_ and d_xy_, d_yz_, p_y_ respectively (Figure S8, Supporting Information). Orbital projected calculations along the M‐Γ‐M/L‐A‐L directions are further performed to compare with the experimental results. We first examine the TaS_2_ band structure and compute the orbital projections under LH/LV polarizations (Figure 3c) to show good agreement with experiments. With LH polarization, the dominant features coming from host TaS_2_ are a hole pocket (Ta d_z2_) above the aforementioned gap and *X*‐shaped bands (S p_z_/p_x_) below. They get strongly suppressed and instead, two electron and hole pockets (S p_y_) are observed in LV polarization.

Based on the selection rules and orbital projections, we further check the parities of the flat band in Mn_1/4_TaS_2_. It is worth noting that there are three sets of flat bands according to the calculation, two of which are located at relatively constant energy while one has noticeable dispersion (Figure 1e and Figure S1, Supporting Information). According to the crystal field analysis, Ta in the H phase TaS_2_ is in trigonal prismatic coordination with local D_3_ _h_ symmetry, while the Mn intercalants occupy the octahedral‐like interstitial sites and have local D_3d_ symmetry, the same as T phase TMD. Both D_3_ _h_ and D_3d_ symmetries have three groups of irreducible representations, thus five degenerate *d* orbitals split into d_z2_, d_x2‐y2_/d_xy_ and d_xz_/d_yz_ respectively due to crystal field splitting.^[^ ^17^, ^18^ ^]^ Among these, the d_z2_ orbitals are more susceptible to NNN hopping in the *z*‐direction, therefore, are relatively more dispersed. In a tight binding simulation (see Note SI, Supporting Information), the flat band position is strongly affected by onsite potentials, therefore the observation of three flat bands in the orbital projected calculation is consistent with the symmetry analysis and tight binding modeling. The d_x2‐y2_/d_xy_ (d_xz_/d_yz_) flat bands are doubly‐degenerate and the two orbitals have opposite parities with respect to the mirror plane, thus polarization‐dependent spectral intensity variation in the ARPES measurements is expected. The overall orbital projected DFT calculation of Mn_1/4_TaS_2_ (Figure 3d) is further examined. Contributions to the flat band observed in ARPES mainly come from Mn d_xz_/d_yz_ and Ta d_z2_/d_x2‐y2_/d_xy_ orbitals, and S p_z_/p_x_/p_y_ spectral weights become more pronounced for flat bands of spin down Mn, due to their lower onsite potentials and stronger hybridization. The calculation shows the same weight distribution of flat bands, where the projected orbitals have stronger spectral intensity at Gamma point under LV polarization and weaker intensity under LH polarization. The result is in good agreement with ARPES measurements and reveals the orbital characters of flat bands in Mn_1/4_TaS_2_.

### 1.3 Flat Bands in Other Intercalated TMDs

The flat band in Mn_1/4_TaS_2_ is identified experimentally by ARPES measurements and theoretically by DFT calculation as well as tight binding modeling. From here, the ubiquitous existence of flat bands in a wider collection of intercalated TMD is examined, and the phenomenon is shown to be generic and generalizable to other TMD families.

**Figure 4:**
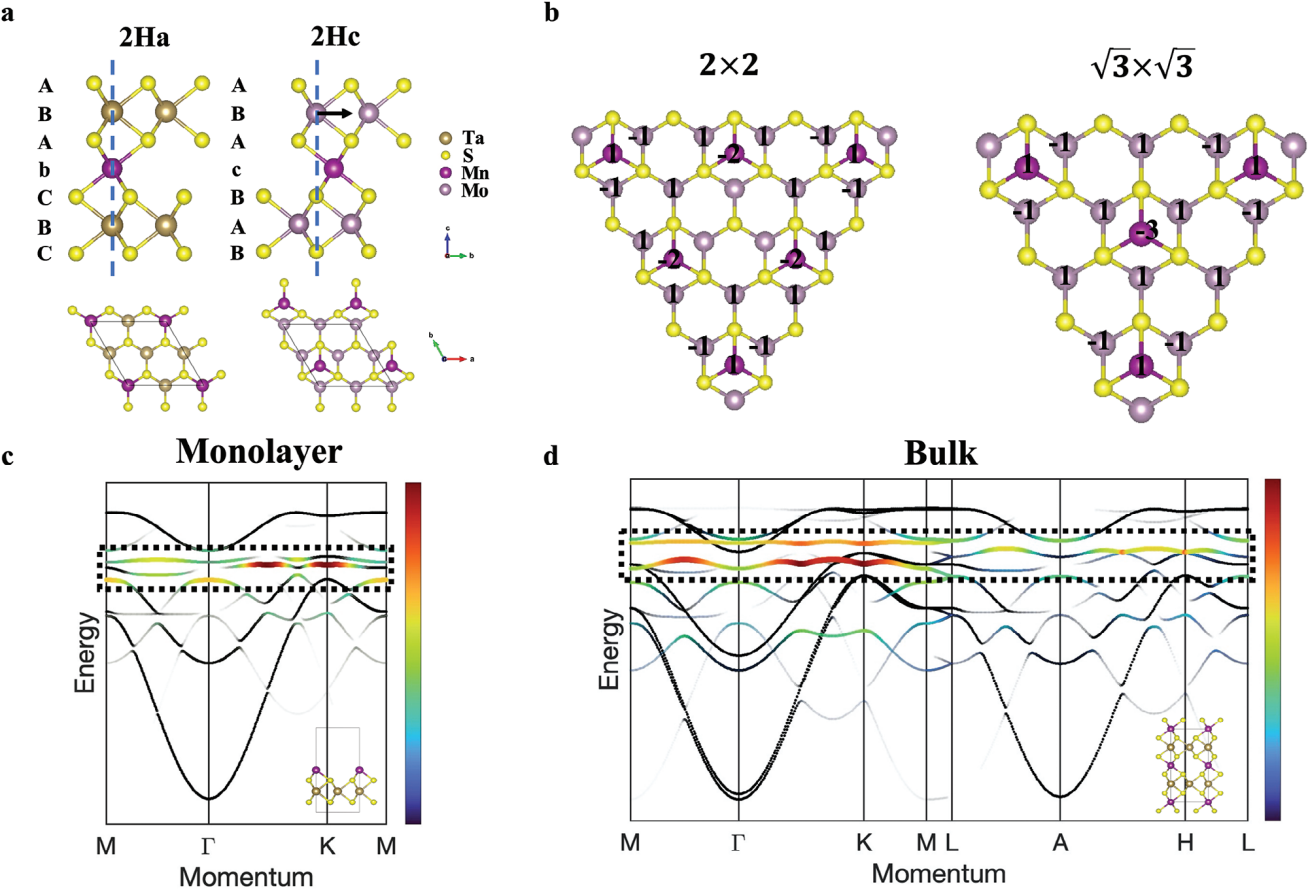
Flat bands in other sparsely intercalated TMD. a) Side and top view of intercalation structure of 2H_a_‐ and 2H_c_‐TMD. The dashed lines mark the interlayer alignment of TM atoms with respect to chalcogen atoms. “A,” “B/b” and “C/c” indicate the layer stacking sequences and intercalant positions. b) Localized states in 2H_c_ structures of (left) 2 × 2 and (right) $\sqrt{3}\;\times \;\sqrt{3}$ with the amplitude/phase of each atom labeled. To simplify the hopping parameters are set the same. c,d) Tight binding band structures in (c) monolayer and (d) bulk intercalated 2H_a_ TMD (same parameters used in Figure S14, Supporting Information). Colors represent the Mn flat bands (indicated by dashed boxes) weight projections. Insets are tight‐binding model structures.

In a TMD with 2H_a_ structure like Mn_1/4_TaS_2_, the transition metal (TM) atoms are aligned in the *c* direction. From STEM results and first principle relaxations, the intercalants in the vdW gap are aligned with the TM atoms in adjacent layers (**Figure** **4**a left panel, stacking ABA“b”CBC…). However, the intercalation can be quite different in 2H_c_‐TMD materials like MoS_2_.^[^ ^19^ ^]^ In the unit cell of a 2H_c_ structure, there is an in‐plane sliding between layers, and the TM atoms of one layer are no longer aligned with those of the next layer, instead aligned with the chalcogen atoms. The intercalants between layers now sit in the hollow sites of the honeycomb‐like TMD lattice (Figure 4a right panel, stacking ABA“c”BAB…). This is more like a buckled and dilute version of Dice lattice, which is a well‐known frustration lattice that can host flat bands.^[^ ^20^, ^21^, ^22^ ^]^ The Dice lattice realization can be achieved in intercalated 2H_c_‐TMD, because intercalants have strong hopping with chalcogen atoms but negligible hopping with TM atoms due to the extension into 3D layered structure.

Although the 2 × 2 supercell tight binding simulation shows the same band dispersion as well as flat band existence in intercalated 2H_a_ and 2H_c_ structures, the underlying localization mechanisms are quite different^[^ ^23^ ^]^ The localized state in the 2H_a_ structure is confined in the trigonal bipyramid structure (Figure 1d), and the wavefunction cancellation happens between the intercalant and its neighboring aligned TM atom through chalcogen atoms. But this becomes more complicated in the 2H_c_ structure (Figure 4b). The environment of an intercalant involves three neighboring TM atoms, and the localized state is geometrically confined in a large triangle that encloses 6 intercalated atoms. The amplitudes and phases of each atom are indicated and they form destructive interference on the chalcogen atoms along the perimeter. This prevents any wavefunction propagation out of the triangle, leading to the electronic localization and flat bands with quenched kinetic energy.

More situations for intercalation concentrations and supercell structures are examined. It is not hard to find that the flat band exists in 2H_a_ structures, independent on the supercell geometry even for random dilute intercalations. This is because the localization only involves the intercalant and its neighboring aligned TM atoms. The situations are quite different and complex for the 2H_c_ structures. Although the flat band still exists in these structures, the geometry of localization is supercell‐dependent. The localized states of $\sqrt{3}\;$×$\;\sqrt{3}$ supercell is presented in Figure 4b and other commonly occurring cells are summarized in Figure S9 (Supporting Information). The 1 × 1 primitive cell is indeed a Dice lattice and other supercells can be viewed as generalized cases. For a *n* × *n* supercell, the geometry of the localized state always consists of a (*n* ^2^ + 1) × (*n* ^2^ + 1) triangle without the very corner TM atoms. Clearly, the localization is more extended in real space with lower intercalation concentration. The triangle orientation is aligned with the host TMD lattice, and the corners always start and end with intercalants. For an arbitrary intercalation ratio forming an ordered supercell in 2H_c_‐TMD, we can always construct such localized triangular electron pockets and achieve flat bands. When there is more than one type of intercalant in one unit cell, or supercells with different sizes are mixed, the localization can also be realized by the superposition of the individual localized pockets (Figure S10, Supporting Information), and flat bands are still present.

We further extend the tight binding simulation to monolayer and bulk intercalated 2H_a_‐TMD models respectively. The monolayer structure (Figure 4c) contains a single TMD layer with 2 × 2 intercalants on the surface in the slab model, while the bulk structure (Figure 4d) has two layers in 2H_a_ stacking with intercalants aligned with TM atoms in the unit cell. The tight binding calculation (with NNN hopping of Ta‐Ta, S‐S, and Ta‐Mn taken into consideration as well as k_z_ dispersion) again reveals the existence of nearly flat bands in both structures. The observed deviation from flatness comes from the not‐perfect cancellation on the S sites in the monolayer case, and from the k_z_‐dependent interlayer hopping along the c direction in the bulk case (see Note SV and Figures S11,S12, Supporting Information). The T phase TMD and other ways of intercalant stacking are further explored (Figure S14, Supporting Information). The nearly dispersionless features are preserved among all categories discussed above, revealing the wide generality of flat band existence in the intercalated TMD family.

## 2 Conclusion

In conclusion, we provide experimental and theoretical evidence for the existence of flat bands in the model intercalated TMD system Mn_1/4_TaS_2_. The weak direct interaction between adjacent Mn atoms, and the destructive interference between Mn and Ta on S contribute to the electron wavefunction localization and vanishing energy dispersion. With tight binding modeling, we further provide a generic way to introduce flat bands into TMD family by intercalation. The position of flat bands on the energy scale can be tuned by varying intercalant species and ratios, to achieve different onsite potentials and crystal field/spin exchange splitting shifts. Moreover, strong spin‐orbital coupling interaction can be introduced from either host TMD compounds or intercalants with d electrons, to explore potential topological features^[^ ^24^ ^]^ Therefore, the criteria for flat bands in such systems comprise 1) dilute intercalation to eliminate direct hopping between intercalants and reduce dispersions from influences like NNN hopping; 2) successful intercalation rather than random substitution or interstitial defects to maintain the destructive interference, and 3) closer onsite potentials of the intercalants and the host transition elements for more ideal electron wavefunction cancellation. In the meantime, the choice of onsite potentials is also a powerful way to adjust the flat band position with respect to the Fermi level. The construction of flat bands provides a unique route for engineering electron correlations in TMDs, and for the systematic exploration and manipulation of the rich properties within this class of materials.

## 3 Experimental Section

### Single Crystal Growth

Single crystals of Mn_1/4_TaS_2_ were grown using the chemical vapor transport (CVT) technique, with iodine added as the transport agent. A mixture of Mn (99.9%), Ta (99.9%), and S (99.9%) with a nominal stoichiometry of Mn_1/4_TaS_2_ was ground and placed into a quartz tube, followed by the introduction of iodine into the mixture. The quartz tube was then evacuated to remove any air or gas. Subsequently, the tube was subjected to a thermal treatment in a gradient from 1260 to 1170 K for 10 days. Upon opening the tube, the obtained crystals were cleaned using supersaturated aqueous solutions of KI for ultrasonication, followed by washing with deionized water and alcohol. The crystals have dimensions of up to several mm in diameter and exhibit a hexagonal morphology.

### Sample Preparation and Characterization

STM measurements were carried out in a commercial Omicron LT‐STM with an ultrahigh vacuum (base pressure better than 1 × 10^−10^ mbar). Single crystal flakes with regular hexagonal shapes were mounted on the STM sample holders with conducting epoxy H21D and heated up to 150° for 15 min. Then ceramic posts were attached on the flakes’ surfaces with epoxy H21D and the samples were heated at 150° for another 45 min. The samples were loaded into STM and cleaved in situ by a wobble stick to expose fresh surfaces for the measurements at 77K (the cleaving method in ARPES was the same as in STM). A tungsten tip was used and calibrated on Au (111) before the measurement. Single‐crystal XRD patterns were collected with a D8‐VENTURE‐XRD diffractometer with a 2‐bounce Ge (022) monochromator and Cu Kα line (*λ* = 0.15418 nm). STEM samples were prepared by focus ion beam (FIB) with a Zeiss Auriga 40 SEM/FIB and cross‐section high‐angle annular dark‐field (HAADF) images were collected with a TFS Spectra Ultra.

### ARPES Measurements

The ARPES measurements were performed at the Quantum Matter Spectroscopy Center (QMSC) beamline at the Canadian Light Source. The samples were cleaved in situ at pressures better than 10^−11^ Torr. The measurements were performed at base temperature of 15 K using a Scienta R4000 hemispherical analyzer equipped with a horizontal entrance slit. Overall angle and energy resolution were better than 0.1° and 23 meV respectively. Photon energy 75 eV was used with varying linear horizontal and linear vertical polarization.

### DFT and Wannier Calculations

All first‐principles DFT calculations were implemented in the QUANTUM ESPRESSO package^[^ ^25^, ^26^ ^]^ using the Perdew–Burke–Ernzerhof exchange‐correlation functional^[^ ^27^ ^]^ A cut‐off energy of 780 eV was used for the plane‐wave basis set and a *k*‐point mesh of 6 × 6 × 4 was applied for the 3D Brillouin zone sampling. The structures were fully relaxed until the residual force on each atom was under 0.01 eV Å‐1. Hubbard *U*  =  2 eV on Mn d orbitals was used for band structure calculations. The Wannier calculations including 98 orbitals (Mn: 3d, Ta: 5d, and S: 2p) were fitted from DFT results with Wannier functions using the Wannier90 package code.^[^ ^28^, ^29^ ^]^ Supercell band unfolding was performed by calculating the spectral weights at each *k*‐point in the primitive Brillouin zone.^[^ ^30^, ^31^ ^]^

### ARPES EDC Peak Fitting

ARPES intensity can be expressed in terms of the product of interaction matrix element *M*, the Fermi‐Dirac distribution function *f* and the single‐particle spectral function:

(1) $$I\left(k,\omega \right)=M\left(k,\omega \right)f\left(\omega \right)A\left(k,\omega \right)$$

The matrix element term *M* can be approximated as a constant and the Fermi–Dirac distribution function *f* describes almost occupied states below the Fermi level at low temperature. Therefore, the intensity is proportional to the single‐particle spectral function *A* which has a Lorentzian function form:

(2) $$A\left(k,\omega \right)=-\frac{1}{\pi }\frac{\Sigma^{\prime \prime }\left(k,\omega \right)}{{\left[\omega -\varepsilon_{0}\left(k\right)-\Sigma^{\prime }\left(k,\omega \right)\right]}^{2}+{\left[\Sigma^{\prime \prime }\left(k,\omega \right)\right]}^{2}}$$

To track the peaks of flat bands in EDCs with a given momentum, a Lorentzian fitting with background is applied:

(3) $$I\;\left(E\right)=\frac{A}{\pi }\;\frac{\Gamma /2}{{\left(E-E_{0}\right)}^{2}+{\left(\Gamma /2\right)}^{2}}+B\left(E\right)$$

where *A* is the amplitude of the spectral function, *E*_0_ is the peak position, Γ is the full width at half maximum (FWHM) of the spectral function and *B*(*E*) is the cubic polynomial background *B* (*E*) = *b*_0_  + *b*_1_ *E* + *b*_2_ *E* ^2^ + *b*_3_ *E* ^3^.

## Conflict of Interest

The authors declare no conflict of interest.

## Supporting information



Supporting Information

## Data Availability

The data that support the findings of this study are available from the corresponding author upon reasonable request.
