# Supplementary material for: Flat Band Generation Through Interlayer Geometric Frustration in Intercalated Transition Metal Dichalcogenides
Source: Small. 2025 Jan 26;21(8):2409535. doi: 10.1002/smll.202409535 (PMC11855231; doi:10.1002/smll.202409535)
Supplement: Supplementary file 1 — Supporting Information [file SMLL-21-2409535-s001.docx]

**Supplementary Notes:**

**SI. Tight binding model for intercalated TMD**

The simplified tight binding structure uses an artificial monolayer which is the building block of Mn_1/4_TaS_2_ unit cell, containing only the chalcogen atoms on one side. This TaS layer is honeycomb-like but not on the same plane, and a 2x2 Mn sublattice is stacked on the chalcogen side. From the TEM measurement and crystal structure analysis, the intercalant’s position is aligned with transition metal atom in 2H_a_ TMD, while in 2H_c_ stacking it is also possible to be in the interstitial site. In the following model the 2x2 Mn are directly aligned with Ta (Fig. S2a). The unit cell has 4 Ta, 4 S and 1 Mn atoms and the Hamiltonian is a 9x9 Hermitian matrix when only s-orbitals are taken into consideration. Here we adopt the lattice gauge and the nearest neighbor hopping model can be written as

$$H=\left( \begin{matrix} \varepsilon_{Ta} & 0 & 0 & 0 & t_{1} & ⅇ^{-ⅈk_{1}}t_{1} & ⅇ^{-ⅈk_{2}}t_{1} & 0 & 0 \\ 0 & \varepsilon_{Ta} & 0 & 0 & t_{1} & t_{1} & 0 & ⅇ^{-ⅈk_{2}}t_{1} & 0 \\ 0 & 0 & \varepsilon_{Ta} & 0 & t_{1} & 0 & t_{1} & ⅇ^{-ⅈk_{1}}t_{1} & 0 \\ 0 & 0 & 0 & \varepsilon_{Ta} & 0 & t_{1} & t_{1} & t_{1} & 0 \\ t_{1} & t_{1} & t_{1} & 0 & \varepsilon_{S} & 0 & 0 & 0 & t_{2} \\ ⅇ^{ⅈk_{1}}t_{1} & t_{1} & 0 & t_{1} & 0 & \varepsilon_{S} & 0 & 0 & ⅇ^{ⅈk_{1}}t_{2} \\ ⅇ^{ⅈk_{2}}t_{1} & 0 & t_{1} & t_{1} & 0 & 0 & \varepsilon_{S} & 0 & ⅇ^{ⅈk_{2}}t_{2} \\ 0 & ⅇ^{ⅈk_{2}}t_{1} & ⅇ^{ⅈk_{1}}t_{1} & t_{1} & 0 & 0 & 0 & \varepsilon_{S} & 0 \\ 0 & 0 & 0 & 0 & t_{2} & ⅇ^{-ⅈk_{1}}t_{2} & ⅇ^{-ⅈk_{2}}t_{2} & 0 & \varepsilon_{Mn} \end{matrix} \right)$$

where $t_{1}$ and $t_{2}$ are the nearest neighbor hopping between Ta-S and Mn-S, $k_{1}$ and $k_{2}$ are phases with $k_{i}=\vec{k}\cdot\vec{r_{i}}$, and $\varepsilon$ denotes different elements’ onsite potentials. Each Mn atom has a Ta symmetrically aligned with respect to the S plane and share the same hopping phases. The differences between these two sites are onsite potentials and hopping strengths. Therefore we can rewrite the Hamiltonian in a new basis by normalizing their states with a unitary transformation $H_{new}=U^{\dagger}HU$. Only sites of Mn and the aligned Ta are modified. Here the unitary matrix U and the new Hamiltonian take the form

$$U=\left( \begin{matrix} \frac{t_{1}}{\sqrt{{t_{1}}^{2}+{t_{2}}^{2}}} & 0 & \frac{t_{2}}{\sqrt{{t_{1}}^{2}+{t_{2}}^{2}}} \\ 0 & I_{7\times7} & 0 \\ -\frac{t_{2}}{\sqrt{{t_{1}}^{2}+{t_{2}}^{2}}} & 0 & \frac{t_{1}}{\sqrt{{t_{1}}^{2}+{t_{2}}^{2}}} \end{matrix} \right)$$

$$H_{new}=$$

$$\left( \begin{matrix} \frac{{{t_{1}}^{2}\varepsilon}_{Ta}+{{t_{2}}^{2}\varepsilon}_{Mn}}{{t_{1}}^{2}+{t_{2}}^{2}} & 0 & 0 & 0 & \sqrt{{t_{1}}^{2}+{t_{2}}^{2}} & ⅇ^{-ⅈk_{1}}\sqrt{{t_{1}}^{2}+{t_{2}}^{2}} & ⅇ^{-ⅈk_{2}}\sqrt{{t_{1}}^{2}+{t_{2}}^{2}} & 0 & \frac{t_{1}t_{2}\left( {\varepsilon_{Mn}-\varepsilon}_{Ta} \right)}{{t_{1}}^{2}+{t_{2}}^{2}} \\ 0 & \varepsilon_{Ta} & 0 & 0 & t_{1} & t_{1} & 0 & ⅇ^{-ⅈk_{2}}t_{1} & 0 \\ 0 & 0 & \varepsilon_{Ta} & 0 & t_{1} & 0 & t_{1} & ⅇ^{-ⅈk_{1}}t_{1} & 0 \\ 0 & 0 & 0 & \varepsilon_{Ta} & 0 & t_{1} & t_{1} & t_{1} & 0 \\ \sqrt{{t_{1}}^{2}+{t_{2}}^{2}} & t_{1} & t_{1} & 0 & \varepsilon_{S} & 0 & 0 & 0 & 0 \\ ⅇ^{ⅈk_{1}}\sqrt{{t_{1}}^{2}+{t_{2}}^{2}} & t_{1} & 0 & t_{1} & 0 & \varepsilon_{S} & 0 & 0 & 0 \\ ⅇ^{ⅈk_{2}}\sqrt{{t_{1}}^{2}+{t_{2}}^{2}} & 0 & t_{1} & t_{1} & 0 & 0 & \varepsilon_{S} & 0 & 0 \\ 0 & ⅇ^{ⅈk_{2}}t_{1} & ⅇ^{ⅈk_{1}}t_{1} & t_{1} & 0 & 0 & 0 & \varepsilon_{S} & 0 \\ \frac{t_{1}t_{2}\left( {\varepsilon_{Mn}-\varepsilon}_{Ta} \right)}{{t_{1}}^{2}+{t_{2}}^{2}} & 0 & 0 & 0 & 0 & 0 & 0 & 0 & \frac{{{t_{1}}^{2}\varepsilon}_{Mn}+{{t_{2}}^{2}\varepsilon}_{Ta}}{{t_{1}}^{2}+{t_{2}}^{2}} \end{matrix} \right)$$

The new effective Hamiltonian replaces the original Ta site by a hybridized state $\frac{1}{t_{2}}|Ta>+\frac{1}{t_{1}}|Mn>$ with effective onsite potential $\frac{{{t_{1}}^{2}\varepsilon}_{Ta}+{{t_{2}}^{2}\varepsilon}_{Mn}}{{t_{1}}^{2}+{t_{2}}^{2}}$ and hopping parameter $\sqrt{{t_{1}}^{2}+{t_{2}}^{2}}$ to S, and the original Mn site by a destructive state $\frac{1}{t_{1}}|Ta>-\frac{1}{t_{2}}|Mn>$ with effective onsite potential $\frac{{{t_{1}}^{2}\varepsilon}_{Mn}+{{t_{2}}^{2}\varepsilon}_{Ta}}{{t_{1}}^{2}+{t_{2}}^{2}}$ and hopping parameter $t_{eff}=\frac{t_{1}t_{2}\left( {\varepsilon_{Mn}-\varepsilon}_{Ta} \right)}{{t_{1}}^{2}+{t_{2}}^{2}}$ only to Ta. It is worth noting that the hopping between Mn and S is eliminated and the remaining hopping is confined between Mn and Ta without k-dispersion. If the onsite potentials of Mn and Ta are the same, the hopping between Mn and Ta becomes zero and a flat band $\varepsilon_{eff}=\frac{{{t_{1}}^{2}\varepsilon}_{Mn}+{{t_{2}}^{2}\varepsilon}_{Ta}}{{t_{1}}^{2}+{t_{2}}^{2}}=\varepsilon_{Mn}$ emerges. The corresponding localized state is then $\frac{1}{t_{1}}|Ta>-\frac{1}{t_{2}}|Mn>$. The Ta and Mn have opposite phases and cancel out on the S sites.

In more general cases, the Mn and Ta onsite potentials are different, the flat band starts to disperse slightly, and the localization is weakened with some wavefunction extension to S sites. To better understand how flat band evolves when the Mn onsite potential $\varepsilon_{Mn}$ changes, we calculate the characteristic polynomial of the new Hamiltonian $f_{k}\left( \lambda\right)=\det\left( H_{new}-\lambda I \right)$. Eigenvalues of the Hamiltonian are roots of $f_{k}\left( \lambda\right)$ where k means the solved $\lambda$ is momentum dependent. By expanding the determinant of $H_{new}-\lambda I$ along the last row, the result can be derived as

$$f_{k}\left( \lambda\right)=\left( \varepsilon_{eff}-\lambda\right){\cdot f}_{k}^{TaS}\left( \lambda\right)-{t_{eff}}^{2}\cdot f_{k}^{HV}\left( \lambda\right)$$

Here $f_{k}^{TaS}\left( \lambda\right)$ is the characteristic polynomial of 2x2 TaS Hamiltonian (which is an 8x8 Hermitian matrix) by deleting the last row and column (the Mn contributions) in $H_{new}-\lambda I$. The eigenvalue spectrum is almost the same as the original 2x2 TaS superlattice and small deviations come from the modified first effective Ta state (Fig. S2b). $f_{k}^{HV}\left( \lambda\right)$ is the characteristic polynomial of the 7x7 Hermitian principal submatrix by further deleting the first row and column (corresponding to the Ta aligned with Mn). The corresponding structure is a honeycomb lattice with periodic 2x2 vacancies (denote as HV - honeycomb vacancy lattice) as shown in Fig. S2c. The HV lattice consists of a Ta kagome sublattice and a defected S hexagonal sublattice. By solving the HV lattice Hamiltonian (Fig. S2d), we find that this HV sublattice contains three flat bands and the dispersionless constant energies are $\lambda_{1}=\varepsilon_{S}, \lambda_{2,3}=\frac{\varepsilon_{S}+\varepsilon_{Ta}}{2}\mp\sqrt{t_{1}^{2}+\left( \frac{\varepsilon_{S}-\varepsilon_{Ta}}{2} \right)^{2}}$, which are the S onsite potential and Ta-S bonding/antibonding energies, respectively. Fig. S2e shows those flat bands’ corresponding localized states with the numbers showing the eigenfunction components’ amplitudes and phases. Now we take a closer look at the whole system $f_{k}\left( \lambda\right)$. The dispersive terms only involve two characteristic polynomials. To eliminate the momentum dispersion, the coefficients should be zero. The first flat band possibility occurs when $t_{eff}=0$ (onsite potentials of Mn and Ta are the same as discussed above) and then one root of $f_{k}\left( \lambda\right)$ is guaranteed to be a constant, $\lambda=\varepsilon_{eff}$. When the Mn/Ta onsite potentials are no longer the same and $t_{eff}\neq0$, dispersionless solutions still exist when $\varepsilon_{eff}=\lambda_{1,2,3}$. It’s not hard to find that while $\varepsilon_{eff}=\lambda_{1,2,3}$, we have $f_{k}\left( \lambda_{i} \right)=-{t_{eff}}^{2}\cdot f_{k}^{HV}\left( \lambda_{i} \right)=0$. $f_{k}\left( {\lambda=\varepsilon}_{eff} \right) is zero and then \varepsilon_{eff}$ is the root of $f_{k}\left( \lambda\right)$ which is a constant. This means flat bands can be preserved over a larger energy window more than $\varepsilon_{Mn}=\varepsilon_{Ta}$. The corresponding localized states are the same as Fig. S2e but with additional destructive states $\frac{1}{t_{1}}|Ta>-\frac{1}{t_{2}}|Mn>$ connected with S at the boundary. Fig. S2f gives the schematic illustration of different onsite potentials and energy levels. These together produce the dominant bands near the Fermi level. While $\varepsilon_{Mn}$ varies, the corresponding $\varepsilon_{eff}$ also varies (dashed line) and it can satisfy or get close to one of the four perfect flat band requirements (black lines), so one nearly flat or segmented flat band (due to band hybridization and avoided crossings) can emerge around the Fermi level. In all these conditions, the flat band position is set by $\varepsilon_{eff}$, which sits between the Mn and Ta onsite potentials. The $\lambda_{1,2,3}$ positions on the other hand mostly vary with the S onsite potential.

In summary, the destructive interference state $\frac{1}{t_{1}}|Ta>-\frac{1}{t_{2}}|Mn>$ contributes to the flat band formation. When their onsite potentials are the same or close, the dispersive hopping term $t_{eff}=\frac{t_{1}t_{2}\left( {\varepsilon_{Mn}-\varepsilon}_{Ta} \right)}{{t_{1}}^{2}+{t_{2}}^{2}}\leq\frac{{\varepsilon_{Mn}-\varepsilon}_{Ta}}{2}$ is negligible, and as a result Mn/Ta can cancel each other on S sites. When the onsite potential difference is getting larger, flat band is still possible when the effective onsite potential matches one of the flat bands of the HV sublattice, and the localization is extended to S sites. Overall, for typical materials, the orbitals of interest normally have their onsite potentials around the Fermi level, and a flat or nearly flat band could readily show up in a wide range of parameters.

**SII. Next nearest neighbor (NNN) hopping in tight binding model**

When NNN hopping is taken into consideration, the Hamiltonian can be written as

$$H=$$

$$\left( \begin{matrix} \varepsilon_{Ta} & \left( 1+ⅇ^{-ⅈk_{1}} \right)t_{Ta} & \left( 1+ⅇ^{-ⅈk_{2}} \right)t_{Ta} & \left( ⅇ^{-ⅈk_{1}}+ⅇ^{-ⅈk_{2}} \right)t_{Ta} & t_{1} & ⅇ^{-ⅈk_{1}}t_{1} & ⅇ^{-ⅈk_{2}}t_{1} & 0 & t_{Mn} \\ \left( 1+ⅇ^{ⅈk_{1}} \right)t_{Ta} & \varepsilon_{Ta} & \left( 1+ⅇ^{ⅈ\left( k_{1}-k_{2} \right)} \right)t_{Ta} & \left( 1+ⅇ^{-ⅈk_{2}} \right)t_{Ta} & t_{1} & t_{1} & 0 & ⅇ^{-ⅈk_{2}}t_{1} & 0 \\ \left( 1+ⅇ^{ⅈk_{2}} \right)t_{Ta} & \left( 1+ⅇ^{-ⅈ\left( k_{1}-k_{2} \right)} \right)t_{Ta} & \varepsilon_{Ta} & \left( 1+ⅇ^{-ⅈk_{1}} \right)t_{Ta} & t_{1} & 0 & t_{1} & ⅇ^{-ⅈk_{1}}t_{1} & 0 \\ \left( ⅇ^{ⅈk_{1}}+ⅇ^{ⅈk_{2}} \right)t_{Ta} & \left( 1+ⅇ^{ⅈk_{2}} \right)t_{Ta} & \left( 1+ⅇ^{ⅈk_{1}} \right)t_{Ta} & \varepsilon_{Ta} & 0 & t_{1} & t_{1} & t_{1} & 0 \\ t_{1} & t_{1} & t_{1} & 0 & \varepsilon_{S} & \left( 1+ⅇ^{-ⅈk_{1}} \right)t_{S} & \left( 1+ⅇ^{-ⅈk_{2}} \right)t_{S} & \left( ⅇ^{-ⅈk_{1}}+ⅇ^{-ⅈk_{2}} \right)t_{S} & t_{2} \\ ⅇ^{ⅈk_{1}}t_{1} & t_{1} & 0 & t_{1} & \left( 1+ⅇ^{ⅈk_{1}} \right)t_{S} & \varepsilon_{S} & \left( 1+ⅇ^{ⅈk_{1}} \right)t_{S} & \left( 1+ⅇ^{-ⅈk_{2}} \right)t_{S} & ⅇ^{ⅈk_{1}}t_{2} \\ ⅇ^{ⅈk_{2}}t_{1} & 0 & t_{1} & t_{1} & \left( 1+ⅇ^{ⅈk_{2}} \right)t_{S} & \left( 1+ⅇ^{-ⅈ\left( k_{1}-k_{2} \right)} \right)t_{S} & \varepsilon_{S} & \left( 1+ⅇ^{-ⅈk_{1}} \right)t_{S} & ⅇ^{ⅈk_{2}}t_{2} \\ 0 & ⅇ^{ⅈk_{2}}t_{1} & ⅇ^{ⅈk_{1}}t_{1} & t_{1} & \left( ⅇ^{ⅈk_{1}}+ⅇ^{ⅈk_{2}} \right)t_{S} & \left( 1+ⅇ^{ⅈk_{2}} \right)t_{S} & \left( 1+ⅇ^{ⅈk_{1}} \right)t_{S} & \varepsilon_{S} & 0 \\ t_{Mn} & 0 & 0 & 0 & t2 & ⅇ^{-ⅈk_{1}}t_{2} & ⅇ^{-ⅈk_{2}}t_{2} & 0 & \varepsilon_{Mn} \end{matrix} \right)$$

where $t_{Ta}$, $t_{S}$ and $t_{Mn}$ are the NNN hopping parameters between Ta-Ta, S-S and Mn-Ta. The dilute intercalation makes sure that Mn-Mn interaction is negligible. The Hamiltonian can be divided into several blocks

$$H=\left( \begin{matrix} H_{Ta} & T_{Ta-S} & T_{Mn-Ta}^{\dagger} \\ T_{Ta-S}^{\dagger} & H_{S} & T_{Mn-S}^{\dagger} \\ T_{Mn-Ta} & T_{Mn-S} & H_{Mn} \end{matrix} \right)$$

where

$$H_{Ta}=\varepsilon_{Ta}I_{4\times4}+t_{Ta}T_{NNN}$$

$$H_{S}=\varepsilon_{S}I_{4\times4}+t_{S}T_{NNN}$$

$$H_{Mn}=\varepsilon_{Mn}$$

$$T_{NNN}=\left( \begin{matrix} 0 & 1+e^{-ik_{1}} & 1+e^{-ik_{2}} & e^{-ik_{1}}+e^{-ik_{2}} \\ 1+e^{ik_{1}} & 0 & 1+ⅇ^{ⅈ\left( k_{1}-k_{2} \right)} & 1+e^{-ik_{2}} \\ 1+e^{ik_{2}} & 1+ⅇ^{-ⅈ\left( k_{1}-k_{2} \right)} & 0 & 1+e^{-ik_{1}} \\ e^{ik_{1}}+e^{ik_{2}} & 1+e^{ik_{2}} & 1+e^{ik_{1}} & 0 \end{matrix} \right)$$

$$T_{Ta-S}=t_{1}\left( \begin{matrix} 1 & e^{-ik_{1}} & e^{-ik_{2}} & 0 \\ 1 & 1 & 0 & e^{-ik_{2}} \\ 1 & 0 & 1 & e^{-ik_{1}} \\ 0 & 1 & 1 & 1 \end{matrix} \right)$$

$$T_{Mn-S}=t_{2}\left( \begin{matrix} 1 & e^{-ik_{1}} & e^{-ik_{2}} & 0 \end{matrix} \right)$$

$$T_{Mn-Ta}=t_{Mn}\left( \begin{matrix} 1 & 0 & 0 & 0 \end{matrix} \right)$$

Fig. S3 gives example band structures of different tight binding parameters as well as NNN hopping. The line width and opacity indicate the contribution after band unfolding from reduced supercell Brillouin zone into primitive Brillouin zone, by considering the Bloch phases of identical atoms in the supercell and calculating the spectral weights of eigenvalues at each k-point^1^. The overall nearly flat features can be observed in these band structures. The deviation from perfect flatness with dispersive and segmented bands come from the NNN hopping and band hybridization with gap opening at avoided band crossings.

**SIII. Set-up of polarization dependent ARPES**

According to the selection rule in linear polarized photoemission, bands having the same parity with respect to the mirror plane can be selectively resolved. Fig. S8a gives the schematic illustration of our ARPES experimental geometry. In the ARPES set-up employed in our measurements, the mirror plane is defined by the photon incident direction and the normal of sample surface, as well as horizontal analyzer slit. When the photon is linear horizontal (linear vertical) polarized, the electric field vector lies in (orthogonal to) the mirror plane, then only orbitals that have even (odd) parity can be resolved. The parity symmetries of d and p orbitals are summarized in Fig. S8b. The selection rule of polarization dependence is well revealed in both the ARPES measurements and DFT calculations of the host TaS_2_ band structures. In Fig. S8c the projected band structures of dominate Ta d orbitals and S p orbitals in linear polarizations are shown. The contribution in linear horizontal polarized ARPES measurement mainly comes from Ta d_z2_/d_xz_ and S p_z_/p_x_ orbitals, while in linear vertical polarization comes from Ta d_yz_ and S p_y_ orbitals.

**SIV. The localized states of flat bands in tight binding model**

In SI we have discussed the tight binding model where intercalants are aligned with transition metal atoms (which is the case in intercalated 2H_a_-TMDs). When the Mn intercalants are not aligned with Ta but interstitial with both Ta and S (correspond to intercalation in 2H_c_-TMDs), the tight binding structure is almost the same as above, except the position of intercalants. The Hamiltonian with nearest neighbor hopping then can be written as

$$H=\left( \begin{matrix} \varepsilon_{Ta} & 0 & 0 & 0 & t_{1} & ⅇ^{-ⅈk_{1}}t_{1} & ⅇ^{-ⅈk_{2}}t_{1} & 0 & 0 \\ 0 & \varepsilon_{Ta} & 0 & 0 & t_{1} & t_{1} & 0 & ⅇ^{-ⅈk_{2}}t_{1} & 0 \\ 0 & 0 & \varepsilon_{Ta} & 0 & t_{1} & 0 & t_{1} & ⅇ^{-ⅈk_{1}}t_{1} & 0 \\ 0 & 0 & 0 & \varepsilon_{Ta} & 0 & t_{1} & t_{1} & t_{1} & 0 \\ t_{1} & t_{1} & t_{1} & 0 & \varepsilon_{S} & 0 & 0 & 0 & t_{2} \\ ⅇ^{ⅈk_{1}}t_{1} & t_{1} & 0 & t_{1} & 0 & \varepsilon_{S} & 0 & 0 & t_{2} \\ ⅇ^{ⅈk_{2}}t_{1} & 0 & t_{1} & t_{1} & 0 & 0 & \varepsilon_{S} & 0 & t_{2} \\ 0 & ⅇ^{ⅈk_{2}}t_{1} & ⅇ^{ⅈk_{1}}t_{1} & t_{1} & 0 & 0 & 0 & \varepsilon_{S} & 0 \\ 0 & 0 & 0 & 0 & t_{2} & t_{2} & t_{2} & 0 & \varepsilon_{Mn} \end{matrix} \right)$$

The hopping phases from Mn are no longer the same as any Ta. By solving the eigenequation of Hamiltonian matrix, we find that the spectra of eigenvalues are the same as before and the difference comes from the eigenvectors. When Mn onsite potential is equal to that of Ta, a flat band located at $\varepsilon_{Ta}$ is observed but the localization is quite different. The eigenstate of this flat band is

$$v=\left( \begin{aligned} t_{2}\left( 1+ⅇ^{ⅈk_{1}}+ⅇ^{ⅈk_{2}} \right) \\ t_{2}\left( -1+ⅇ^{ⅈk_{2}}-ⅇ^{2ⅈk_{1}}+ⅇ^{ⅈk_{1}+ⅈk_{2}} \right) \\ t_{2}\left( -1+ⅇ^{ⅈk_{1}}-ⅇ^{2ⅈk_{2}}+ⅇ^{ⅈk_{1}+ⅈk_{2}} \right) \\ t_{2}\left( ⅇ^{ⅈk_{1}}+ⅇ^{ⅈk_{2}}-ⅇ^{2ⅈk_{1}}-ⅇ^{2ⅈk_{2}} \right) \\ 0 \\ 0 \\ 0 \\ 0 \\ t_{1}\left( 1-2ⅇ^{ⅈk_{1}}-2ⅇ^{ⅈk_{2}}+ⅇ^{2ⅈk_{1}}+ⅇ^{2ⅈk_{2}}-2ⅇ^{ⅈk_{1}+ⅈk_{2}} \right) \end{aligned} \right)$$

in the form of finite sum of the Bloch phases. By applying Fourier transformation on the eigenvector^2^, we can get the localized geometry of flat band in real space. The electronic localization is confined in a large triangle involving 6 Mn atoms and 15 Ta atoms as shown in the main text (Fig. 4b). The wavefunction on S sites are cancelled everywhere along the perimeter. Similarly, when there is onsite potential difference between Mn and Ta, the localization starts to extend to S sites. Fig. S10a gives an example of the flat band’s localized state when onsite potentials of Mn and Ta are different. Here the effective onsite potential $\varepsilon_{eff}$ of Mn and Ta is set to be equal to that of S. Compared with the localization shown in Fig. 4b, the triangular pocket involves addition S atoms inside with nonzero amplitudes. These S sites cancel each other on nearby Ta, and contribute to the electronic localization together with Mn/Ta.

We have discussed the real space localization of flat bands in the most common ordered and dilute supercell intercalation positions. For other complicated intercalation cases, the flat bands can be achieved by the superposition of individual localized pockets. For example, when there are 2 Mn atoms (Fig. S10b) in 2x2 interstitial intercalation (works for aligned stacking as well), the Hamiltonian can be written as

$$H=$$

$$\left( \begin{matrix} \varepsilon_{Ta} & 0 & 0 & 0 & t_{1} & ⅇ^{-ⅈk_{1}}t_{1} & ⅇ^{-ⅈk_{2}}t_{1} & 0 & 0 & 0 \\ 0 & \varepsilon_{Ta} & 0 & 0 & t_{1} & t_{1} & 0 & ⅇ^{-ⅈk_{2}}t_{1} & 0 & 0 \\ 0 & 0 & \varepsilon_{Ta} & 0 & t_{1} & 0 & t_{1} & ⅇ^{-ⅈk_{1}}t_{1} & 0 & 0 \\ 0 & 0 & 0 & \varepsilon_{Ta} & 0 & t_{1} & t_{1} & t_{1} & 0 & 0 \\ t_{1} & t_{1} & t_{1} & 0 & \varepsilon_{S} & 0 & 0 & 0 & t_{2} & {ⅇ^{-ⅈk_{1}}t}_{2} \\ ⅇ^{ⅈk_{1}}t_{1} & t_{1} & 0 & t_{1} & 0 & \varepsilon_{S} & 0 & 0 & t_{2} & t_{2} \\ ⅇ^{ⅈk_{2}}t_{1} & 0 & t_{1} & t_{1} & 0 & 0 & \varepsilon_{S} & 0 & t_{2} & 0 \\ 0 & ⅇ^{ⅈk_{2}}t_{1} & ⅇ^{ⅈk_{1}}t_{1} & t_{1} & 0 & 0 & 0 & \varepsilon_{S} & 0 & t_{2} \\ 0 & 0 & 0 & 0 & t_{2} & t_{2} & t_{2} & 0 & \varepsilon_{Mn} & 0 \\ 0 & 0 & 0 & 0 & {ⅇ^{ⅈk_{1}}t}_{2} & t_{2} & 0 & t_{2} & 0 & \varepsilon_{Mn} \end{matrix} \right)$$

Here the last two rows and columns are hopping of Mn_A_ and Mn_B_ sites. Let $H_{A}$ and $v_{A}$ be the Hamiltonian and localized eigenstate by deleting Mn_B_, then $H$ can be expressed as

$$H=\left( \begin{matrix} H_{A} & P^{\dagger} \\ P & \varepsilon_{Mn} \end{matrix} \right)$$

where $P=\left( \begin{matrix} 0 & 0 & 0 & 0 & {ⅇ^{ⅈk_{1}}t}_{2} & t_{2} & 0 & t_{2} & 0 \end{matrix} \right)$ is the hopping between Mn_B_-Ta, Mn_B_-S and Mn_B_- Mn_A_, with only the Mn_B_-S hopping allowed. It’s not hard to find that the eigenvector of $H$ can be obtained by adding 0 at Mn_B_ site in $v_{A}$ (localization is shown in Fig. S10c)

$$H v_{A}^{'}=\left( \begin{matrix} H_{A} & P^{\dagger} \\ P & \varepsilon_{Mn} \end{matrix} \right)\left( \begin{matrix} v_{A} \\ 0 \end{matrix} \right)=\left( \begin{matrix} {H_{A} v}_{A} \\ P v_{A} \end{matrix} \right)=\left( \begin{matrix} {H_{A} v}_{A} \\ 0 \end{matrix} \right)=E v_{A}^{'}$$

This is because $v_{A}$ is zero at S sites due to the destructive cancellation while $P$ is only non-zero at S sites connected to Mn_B_. As a result, $P v_{A}$ is equal to zero. On the other hand, because all S sites are zero due to destructive interference, they have no hopping contributions to Mn_B_ sites, so we can add 0 at Mn_B_ site in $v_{A}$ as the eigenstate and localization can be kept in this mixed structure. This gives one of the eigenvectors of $H$, and the localization corresponds to the case of single intercalant atom in the unit cell. These are still valid when Mn_A_ and Mn_B_ sites are swapped, which means $v_{B}^{'}$ constructed by the same way (add 0 at Mn_A_ site in $v_{B}$) is the eigenvector as well. These two eigenstates have the same eigenvalues, therefore any superposition of the two individual localized pockets $\alpha v_{A}^{'}+\beta v_{B}^{'}$ is still the eigenvector of $H$ and the flat bands are doubly degenerate.

When $v_{A}$ has finite non-zero values at S sites like Fig. S10a shows, corresponding to the $\varepsilon_{eff}=\varepsilon_{S}$ solution, the equality $P v_{A}=0$ still holds. We can view this localization more clearly when we put Mn_B_ at the interstitial center of any hexagon left in the structure shown in Fig. S10a. The sum of hopping from neighboring S is always zero, in agreement with the equality $P v_{A}=0$. This makes sure that the individual localized state of each intercalant can still keep localization with additional intercalant in the unit cell. When theses intercalants are identical (having same tight binding parameters), the eigenvector is the arbitrary superposition of each individual localized state which is a consequence of flat bands degeneracy. When they are different atom species but satisfy their own dispersionless requirements, the degeneracy no longer exists and there will be multiple flat bands.

Another case is that the intercalation has supercells with different sizes mixed. We can denote these supercells as A and B sublattices (similar to the description of single intercalant atom A and B above), then the same method can be applied. For example, given an intercalation with 2x2 and $\sqrt{7}\times\sqrt{7}$ supercells mixed, we can find a lager common supercell that can enclose both types of intercalants (a 14x14 supercell here), and then construct the supercell Hamiltonian. Because flat bands still stay dispersionless after supercell band folding, the Hamiltonian can be divided into A and B blocks following the same way:

$$H=\left( \begin{matrix} H_{A} & P^{\dagger} \\ P & \varepsilon_{B} \end{matrix} \right)$$

where $H_{A}$ is the Hamiltonian with only one type intercalation, $\varepsilon_{B}$ is the hopping matrix among sublattice B and is diagonal with onsite potentials only due to dilute intercalation, and $P$ is the hopping between sublattice B with S (zero with Ta and sublattice A). Again the equality $P v_{A}=0$ is valid because all S sites are zero due to destructive interference, or the nonzero S wavefunctions cancel each other on neighboring sites, therefore $v_{A\left( B \right)}^{'}$ with zero at sublattice B(A) sites in $v_{A\left( B \right)}$ is the eigenvector of the flat band. Here the individual localized states are the triangular pockets summarized in main text of supercells with different sizes. As a result, real space localization can be achieved with degenerate or multiple flat bands depending on the intercalant species.

**SV. Generalized tight binding model and the kz dependence of the flatness**

Here we generalize tight binding model to the real structures of monolayer and bulk intercalated TMDs respectively. The monolayer TaS_2_ structure with a layer of 2x2 Mn on top is shown in Fig. S11a. Compared with the simplified TaS model discussed above, there is an additional S layer stacked on the other side of Ta and aligned with the previous S layer. The Hamiltonian then has one more block and is a 13x13 Hermitian matrix

$$H=\left( \begin{matrix} H_{Ta} & T_{Ta-S} & T_{Ta-S} & T_{Mn-Ta}^{\dagger} \\ T_{Ta-S}^{\dagger} & H_{S\left( bottom \right)} & T_{S-S} & 0 \\ T_{Ta-S}^{\dagger} & T_{S-S}^{\dagger} & H_{S\left( top \right)} & T_{Mn-S}^{\dagger} \\ T_{Mn-Ta} & 0 & T_{Mn-S} & H_{Mn} \end{matrix} \right)$$

where $T_{S-S}=t_{s}I_{4\times4}$ is the interaction between top and bottom S layers. Because of the absence of Mn layer on the other side to cancel out on S, the strictly flat band disappears and starts to disperse slightly instead. Fig. S11a gives the example band structure with same parameters in Fig. S3d. The flat band located at $\varepsilon_{Ta}$ becomes segmented due to avoided band crossing and hybridization.

The bulk structure has two TaS_2_ layers with a relative 180-degrees rotation. The Hamiltonian can be written as

$$H=\left( \begin{matrix} H_{mono} & 0 & T_{bottom}^{\dagger} \\ 0 & H_{mono}^{*} & T_{top}^{\dagger} \\ T_{bottom} & T_{top} & H_{Mn} \end{matrix} \right)$$

where $H_{mono}$ is the Hamiltonian of bottom TaS_2_ monolayer and takes the form

$$H_{mono}=\left( \begin{matrix} H_{Ta} & T_{Ta-S} & T_{Ta-S} \\ T_{Ta-S}^{\dagger} & H_{S} & T_{S-S} \\ T_{Ta-S}^{\dagger} & T_{S-S}^{\dagger} & H_{S} \end{matrix} \right)$$

. $H_{mono}^{*}$ is the Hamiltonian of top TaS_2_ monolayer and is the conjugate of $H_{mono}$, because of the relative rotation and opposite lattice vectors. $H_{Mn}$, $T_{top}$ and $T_{bottom}$ are Mn onsite potentials and hopping with adjacent TaS_2_ layers and they take the form

$$H_{Mn}=\left( \begin{matrix} \varepsilon_{Mn\left( bottom \right)} & 0 \\ 0 & \varepsilon_{Mn\left( top \right)} \end{matrix} \right)$$

$$T_{bottom}=\left( \begin{aligned} \begin{matrix} t_{Mn} & 0 & 0 & 0 & t_{2} & {ⅇ^{-ⅈk_{1}}t}_{2} & {ⅇ^{-ⅈk_{2}}t}_{2} & 0 & 0 & 0 & 0 & 0 \end{matrix} \\ \begin{matrix} t_{Mn} & 0 & 0 & 0 & 0 & 0 & 0 & 0 & t_{2} & {ⅇ^{-ⅈk_{1}}t}_{2} & {ⅇ^{-ⅈk_{2}}t}_{2} & 0 \end{matrix} \end{aligned} \right)$$

$$T_{top}=\left( \begin{aligned} \begin{matrix} {e^{-i(k_{1}+k_{2}+k_{3})}t}_{Mn} & 0 & 0 & 0 & 0 & 0 & 0 & 0 & e^{-i(k_{1}+k_{2}+k_{3})}t_{2} & {e^{-i(k_{2}+k_{3})}t}_{2} & {e^{-i(k_{1}+k_{3})}t}_{2} & 0 \end{matrix} \\ \begin{matrix} e^{-i(k_{1}+k_{2})}t_{Mn} & 0 & 0 & 0 & e^{-i(k_{1}+k_{2})}t_{2} & {ⅇ^{-ⅈk_{2}}t}_{2} & {ⅇ^{-ⅈk_{1}}t}_{2} & 0 & 0 & 0 & 0 & 0 \end{matrix} \end{aligned} \right)$$

In this model the interlayer hopping is realized by Mn and the direct interaction between TaS_2_ layers is neglected. The band structure is given in Fig. S11b. The bulk 3D structure contains a repeating cell with “Mn-S_top_-Ta-S_bottom_-Mn-…” which has an interlayer hopping. To have destructive interference on S, Mn and Ta will have alternating phases along the out-of-plane direction. Although there are flat bands at $k_{z}=0$ plane, but the localization is two dimensional and extended along out-of-plane direction. Therefore they have k_z_ dispersion and become slightly dispersive when interlayer interaction is taken into consideration (Fig. S12). This is consistent with DFT calculations (Fig. S13) that one of the flat bands at -1.23 eV can get dispersive to some extent at different k_z_, which is a consequence of the delocalization along the out-of-plane direction.

The Hamiltonian of T phase TMD in main text is constructed by the same way, and all the tight binding parameters used in Fig. 4 and Fig. S14 are $\varepsilon_{Ta}=0$eV, $\varepsilon_{S}=-6$eV, $\varepsilon_{Mn}=-2$eV, $t_{1}=-5$eV, $t_{2}=-3$eV, $t_{Ta}=-3$eV, $t_{S}=-2$eV and $t_{Mn}=-2$eV.

**Supplementary Table:**

| EDC | Cut 1 | | Cut 2 | | Cut 3 | | Cut 4 | |
| --- | --- | --- | --- | --- | --- | --- | --- | --- |
| Momentum ($Å$^-1^) | $\bar{\Gamma}$ | $\bar{M}$ | $\bar{\Gamma}$ | $\bar{K}$ | 0.0943785 | $\bar{K}$ | 0.093 | 1.019 |
| Amplitude $A$ | 90.600 | 936.799 | 3308.56 | 2639.04 | 765.583 | 772.457 | 1130.3 | 4423.91 |
| Position $E_{0}$ (eV) | -1.174 | -1.265 | -1.213 | -1.220 | -1.193 | -1.205 | -1.214 | -1.254 |
| FWHM $\Gamma$ (eV) | 0.448 | 0.938 | 0.955 | 0.830 | 0.889 | 0.956 | 0.653 | 0.922 |
| $b_{0}$ | 1220.18 | -451.861 | 5336.47 | 5793.19 | 1890.5 | 1769.46 | 2489.29 | 5853.67 |
| $b_{1}$ (eV^-1^) | 2510.94 | -2063.22 | 10036.9 | 10152.8 | 3994.2 | 4075.93 | 2793.03 | 7689.96 |
| $b_{2}$ (eV^-2^) | 1595.09 | -2968.63 | 2081.86 | 1915.49 | 2015.54 | 2398.32 | -1296.33 | -2496.84 |
| $b_{3}$ (eV^-3^) | 234.36 | -1139.09 | -1274.14 | -1309.38 | 157.148 | 358.555 | -1492.33 | -2940.04 |

Table. S1: Fitting parameters of the flat band peaks in EDCs at high symmetry points.

**Supplementary Figures:**


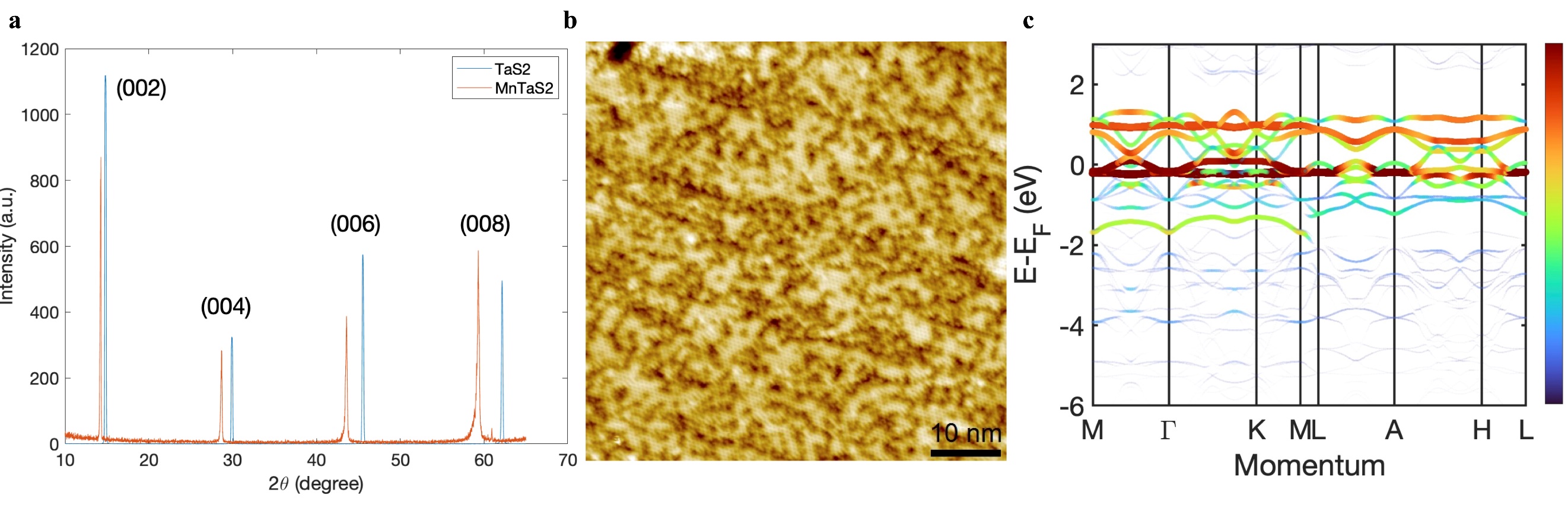


Fig. S1: Crystal characterization and DFT band structure calculation. **a**, XRD measurements of TaS2 and Mn1/4TaS2 single crystals. Calculated out-of-plane lattice constants are 12.46 $Å$ for Mn_1/4_TaS_2_ and 11.94 $Å$ for TaS_2_, respectively. The shift to smaller angles is a direct consequence of the larger interlayer spacing with extra atoms. **b**, STM atomic resolution on Mn-terminated surface (V_bias_ = 1 V, I_set_ = 0.5 A). Scale bar is 10 nm. **c**, DFT band structure of Mn_1/4_TaS_2_ without spin polarization. Line width and color represent Mn weight projection.


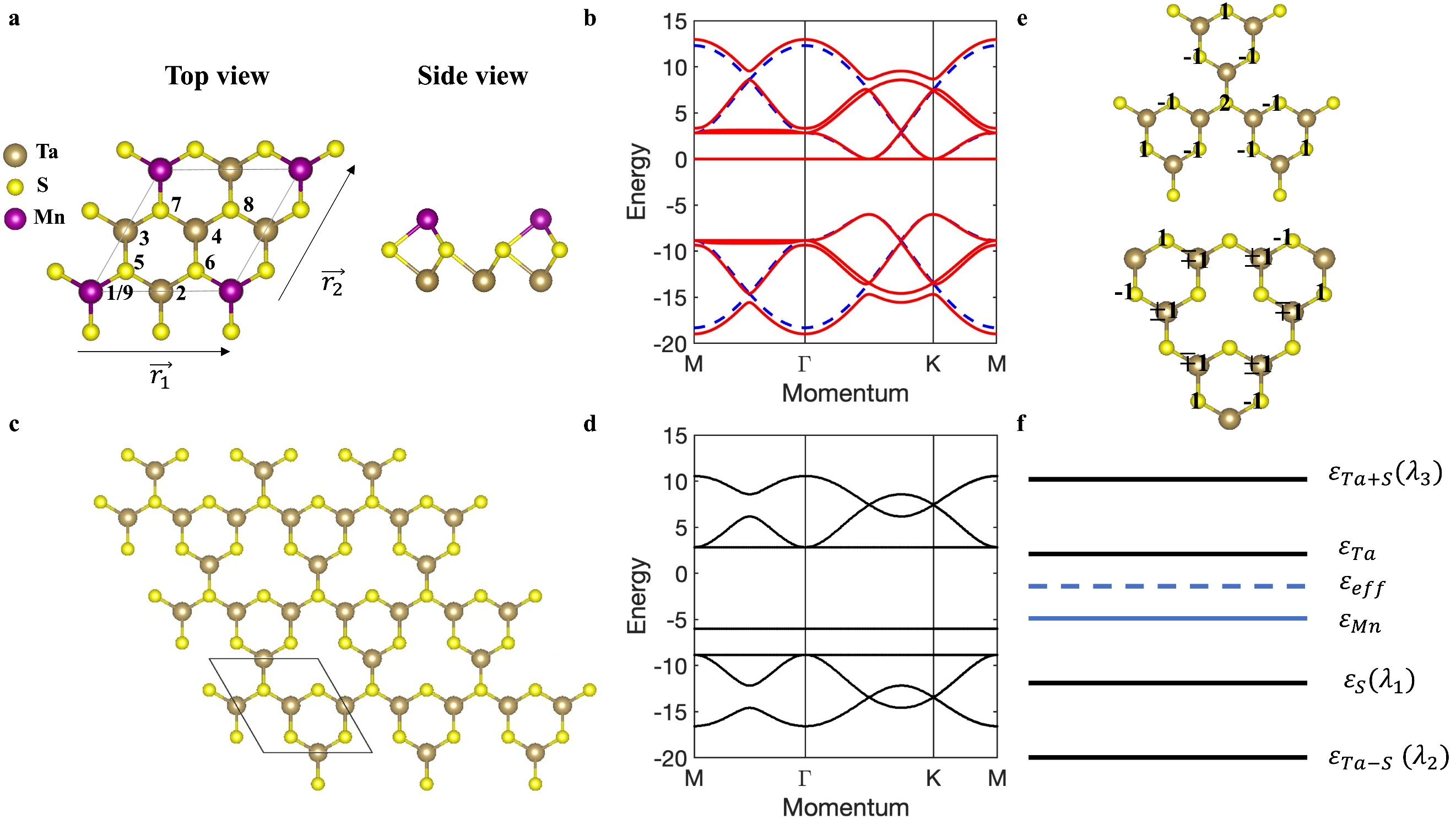


Fig. S2: Tight binding model for intercalated TMD. **a**, Side and top view of the simplified tight binding structure. $\vec{r_{1}}$ and $\vec{r_{2}}$ are unit cell lattice vectors used for the hopping phase, and numbers near atoms indicate their positions (rows and columns) in the Hamiltonian. **b**, Band structures of 2x2 TaS (blue dashed line) and intercalated Mn_1/4_TaS (red solid line). **c**, Structure of honeycomb lattice with periodic 2x2 vacancies (HV lattice). The two sites in the unit cell can be two different atoms. **d**, Band structure of the HV lattice. **e**, Localized states of flat bands in **d**. The upper one corresponds to the solution $\lambda_{1}$($\varepsilon_{S}$) and the lower one corresponds to the solution $\lambda_{2,3}$. **f**, Schematic illustration of different onsite potentials and energy levels. The tight binding parameters used here and in Fig. 1d are $onsite=\left( \varepsilon_{Ta},\varepsilon_{S},\varepsilon_{Mn} \right)=(0, -6, 0)$ eV and ${t=(t}_{1},t_{2})=(-5, -3)$ eV.


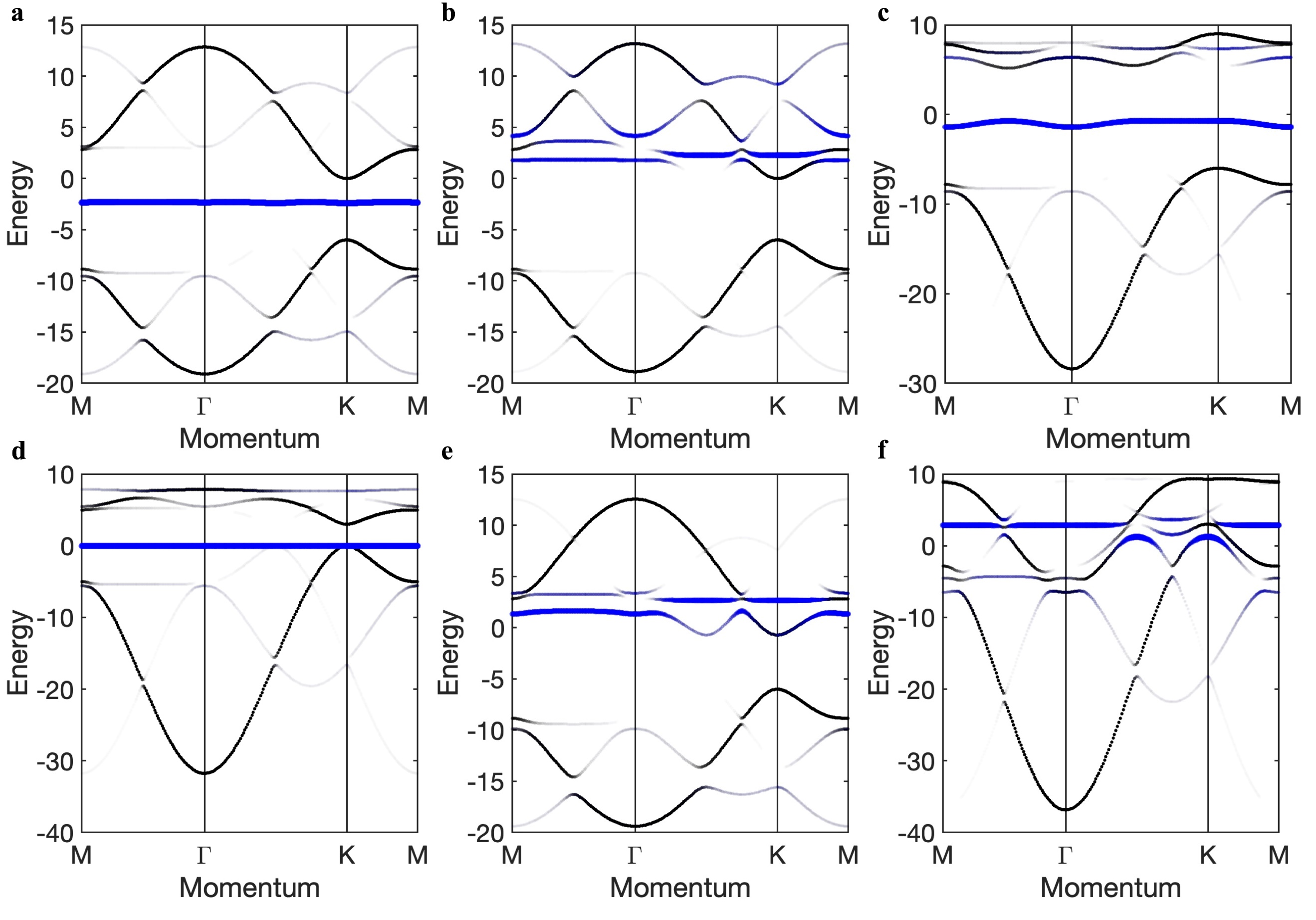


Fig. S3: Band structures with different tight binding parameters. **a**,**b**, Nearly flat bands when the onsite potential of Mn is different from that of Ta. The line width and opacity indicate the spectral weight after band unfolding, and the blue curves represent Mn flat bands. Parameters used are **a** $onsite=(0, -6, -3)$ eV and **b** $onsite=(0, -6, 3)$ eV with hopping $t=(-5, -3)$ eV. **c**-**f**, Nearly flat bands when NNN hopping is taken into consideration. The NNN hopping parameters $t_{NNN}=(t_{Ta},t_{S},t_{Mn})$ are **c** $t_{NNN}=(-3, 0, 0)$ eV, **d** $t_{NNN}=(0, -3, 0)$ eV, **e** $t_{NNN}=(0, 0, -3)$ eV and **f** $t_{NNN}=(-3, -3, -3)$ eV.


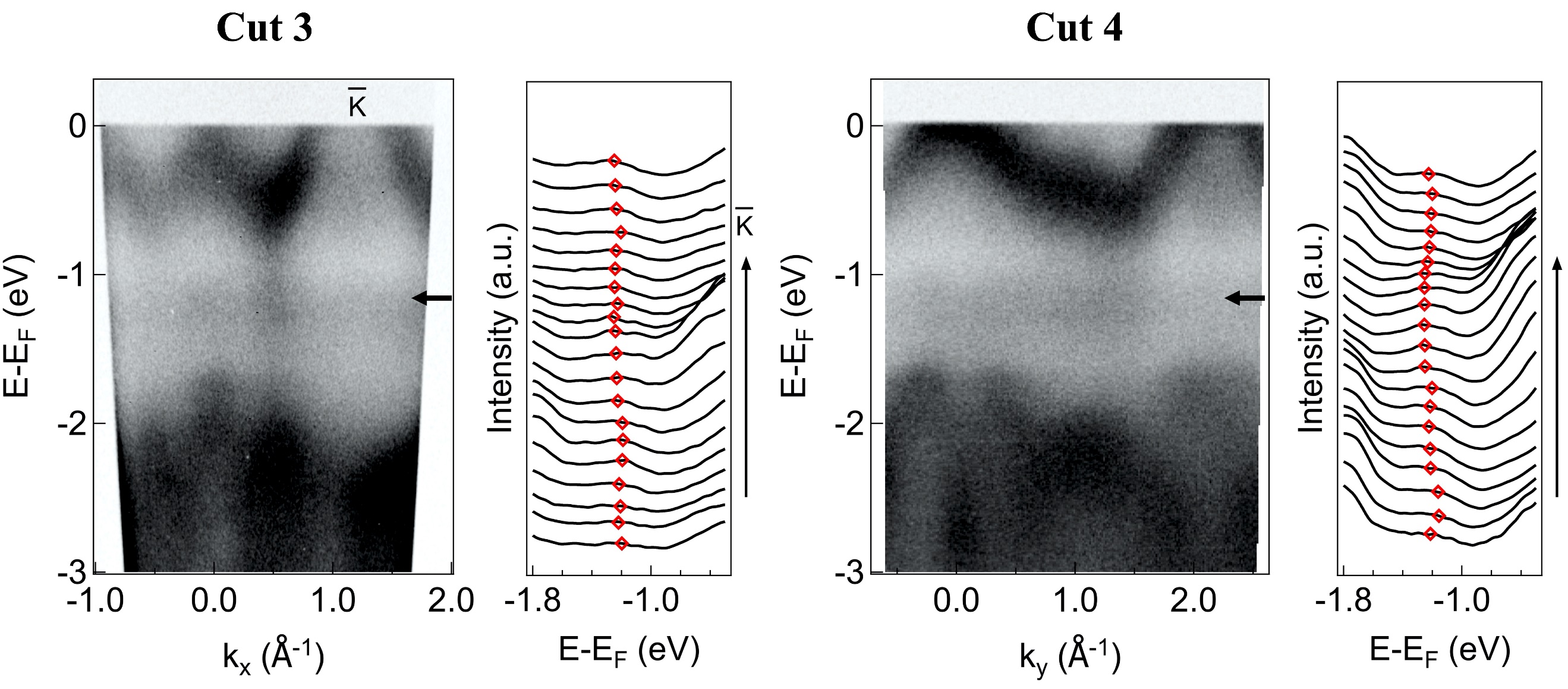


Fig. S4: ARPES spectra taken across high-symmetry points with corresponding EDCs. The directions in (left) Cut 3 and (right) Cut 4 are shown in Fig. 2a. Black arrows mark the flat band energy positions, and red rhombus dots track the flat band peaks in EDCs.


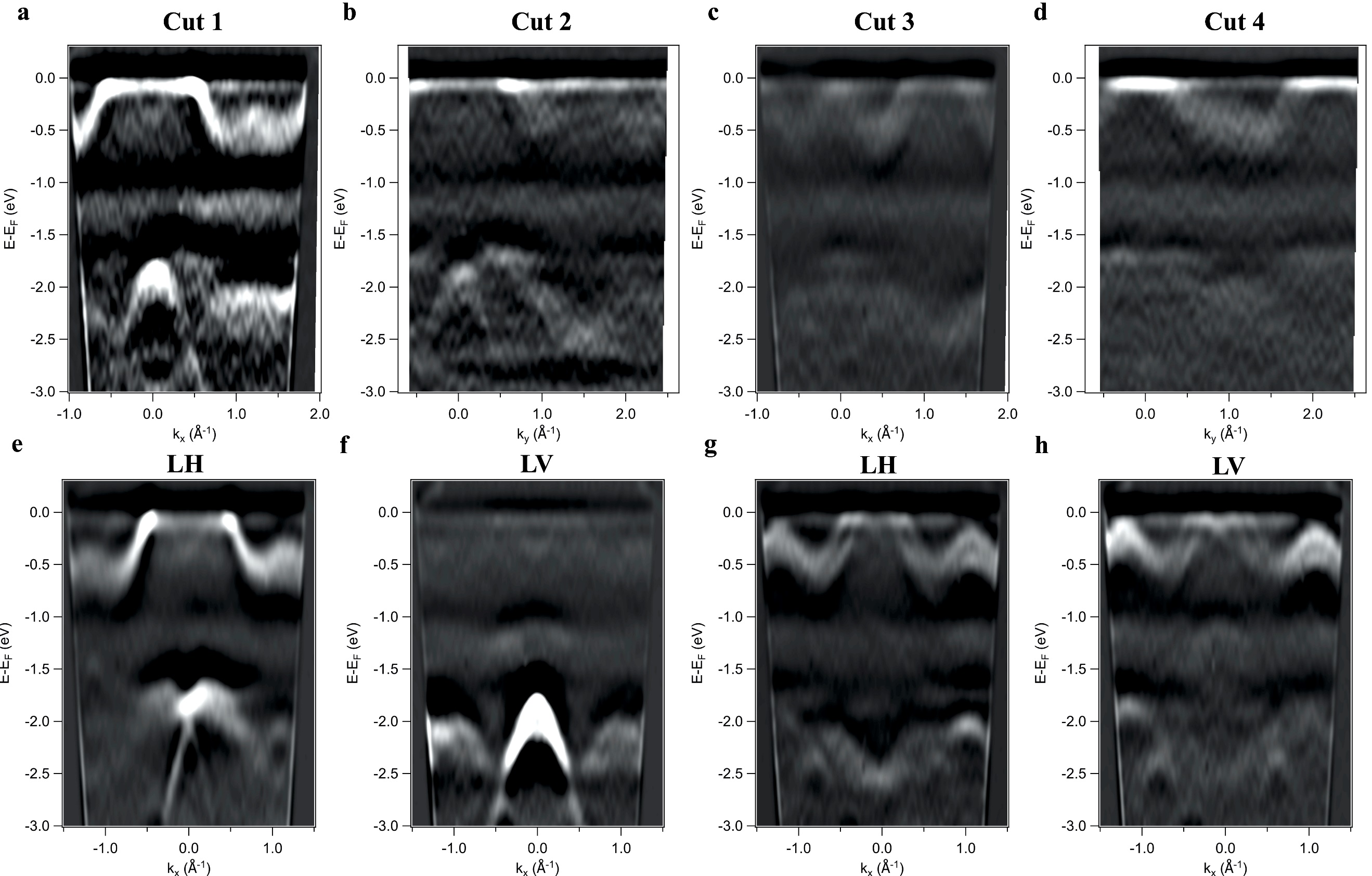


Fig. S5: Second derivative plots of ARPES spectra. **a**-**d**, Plots of Cut 1-4 shown in Fig. 2b and 2c, and Fig. S4. **e**,**f**, Plots of spectra along $\bar{M}-\bar{\Gamma}-\bar{M}$ direction under LH/LV polarizations shown in Fig. 3a. **g**,**h**, Plots of spectra along $\bar{K'}-\bar{K}-\bar{K'}$ direction under LH/LV polarizations shown in Fig. S6. All of them show a clear flat band.


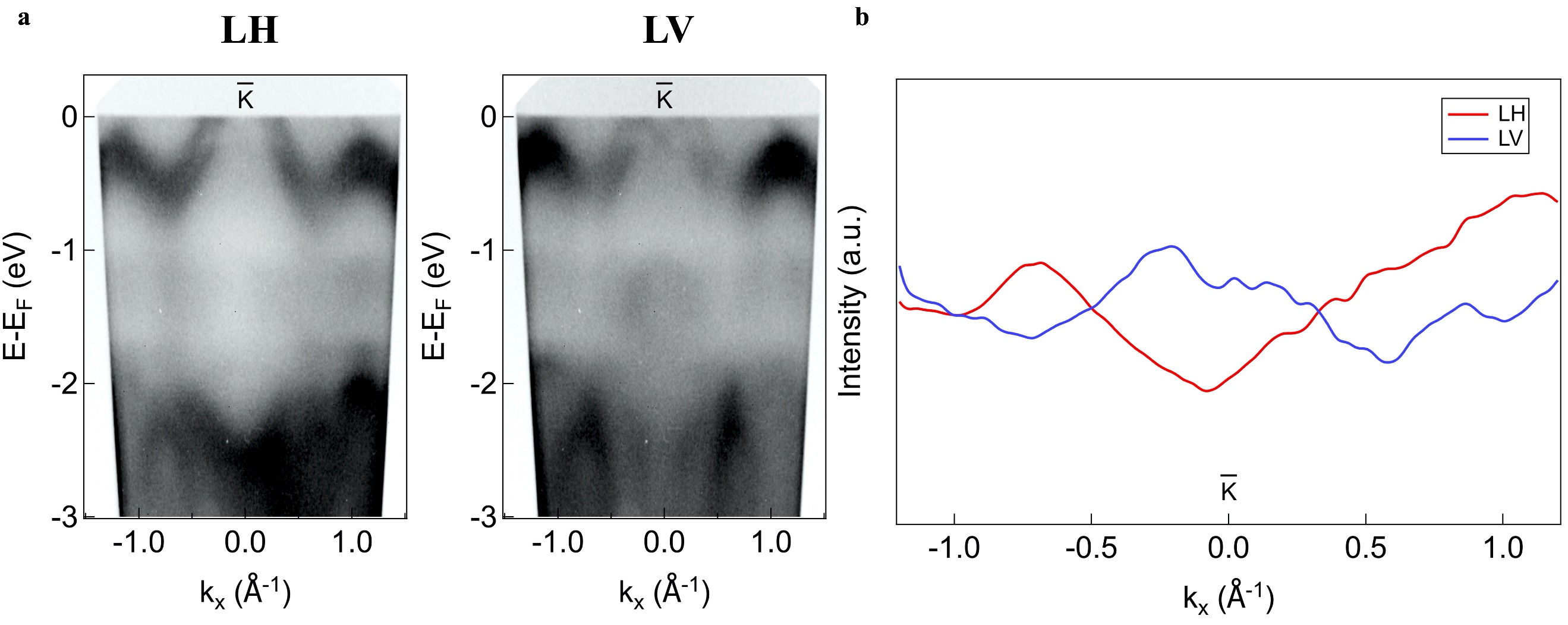


Fig. S6: Polarization dependent ARPES measurements across K point. **a**, ARPES spectra measured along $\bar{K'}-\bar{K}-\bar{K'}$ direction with (left) LH and (right) LV polarizations. **b**, MDCs at the flat band position (integrated over an energy range of 40 meV around -1.23 eV) in (red) LH and (blue) LV polarizations.


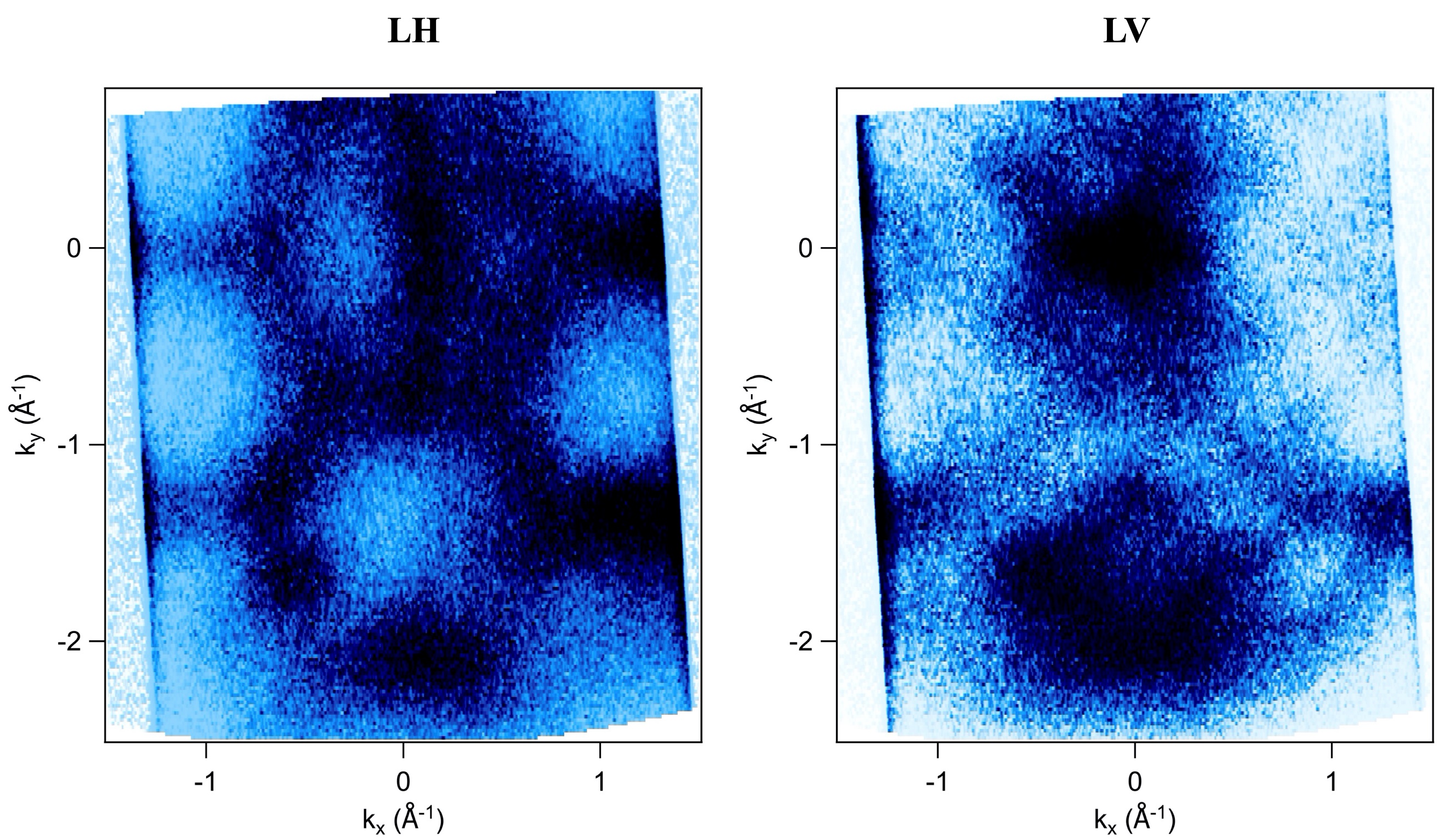


Fig. S7: Constant energy contour mapping at -1.23 eV where flat band is observed under linear horizontal (LH, left) and linear vertical (LV, right) polarizations.


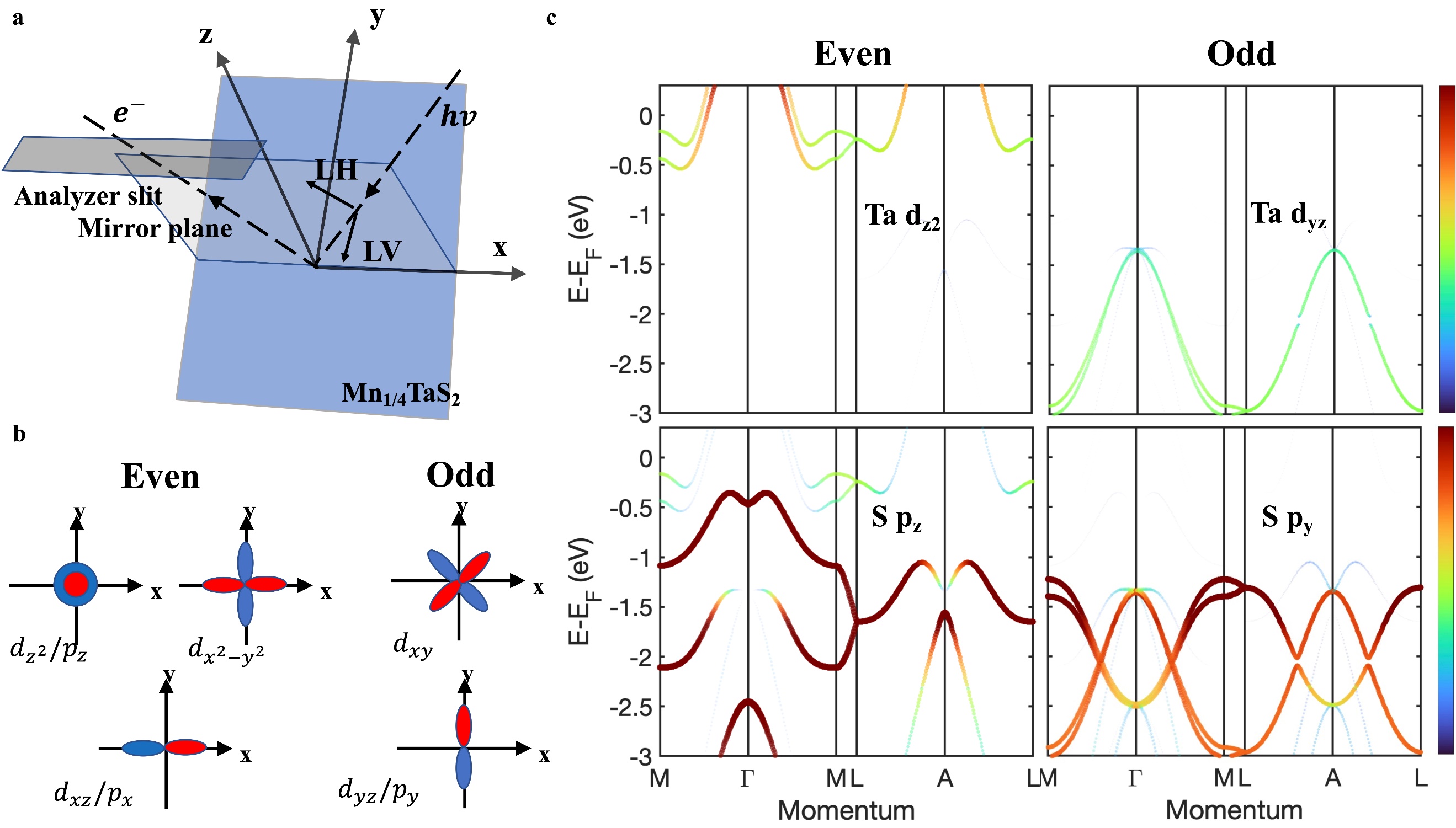


Fig. S8: Set-up of polarization dependent ARPES. **a**, Schematic illustration of ARPES experimental set-up. **b**, Parity symmetries of each d and p orbitals. The d_xz_ (d_yz_) orbital is equivalent to p_x_ (p_y_) orbital. **c**, Dominant orbital projected band structures of TaS_2_. The line width and color depth represent projected orbital contributions in LH/LV polarizations.


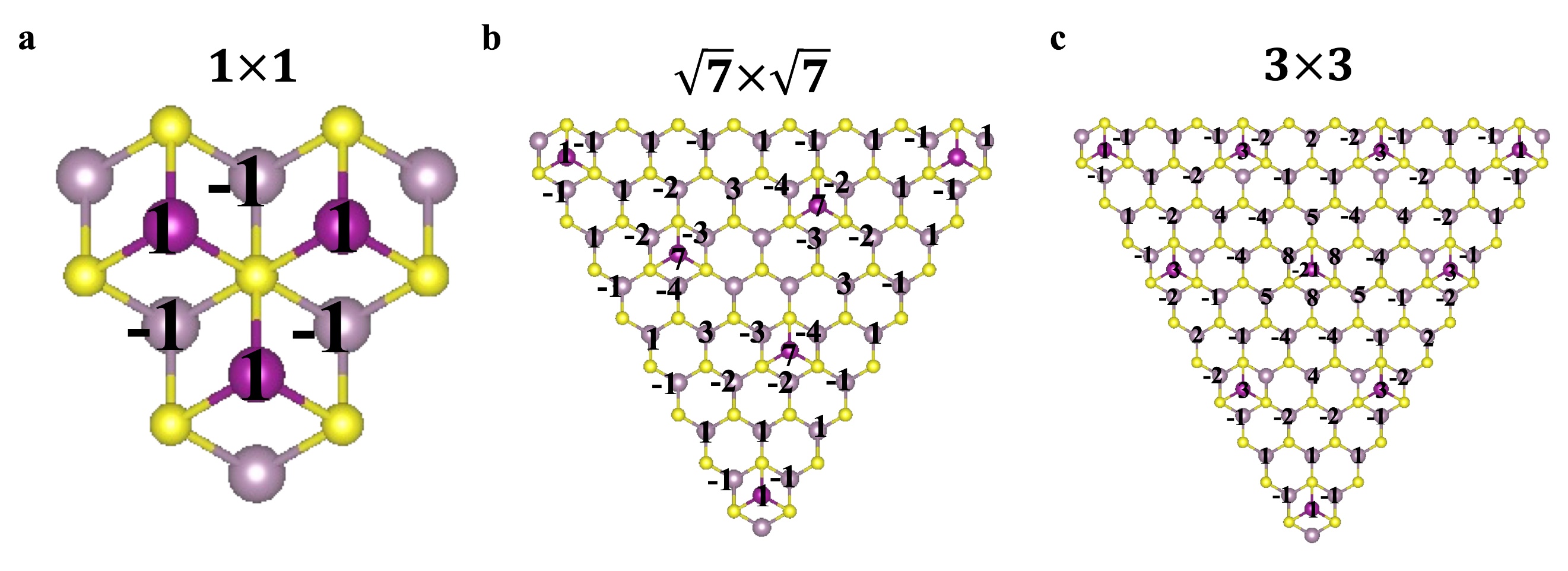


Fig. S9: Localized states of flat bands in **a** 1×1, **b** $\sqrt{7}$×$\sqrt{7}$ and **c** 3×3 supercell intercalated structures. The amplitude/phase of each atom is labeled


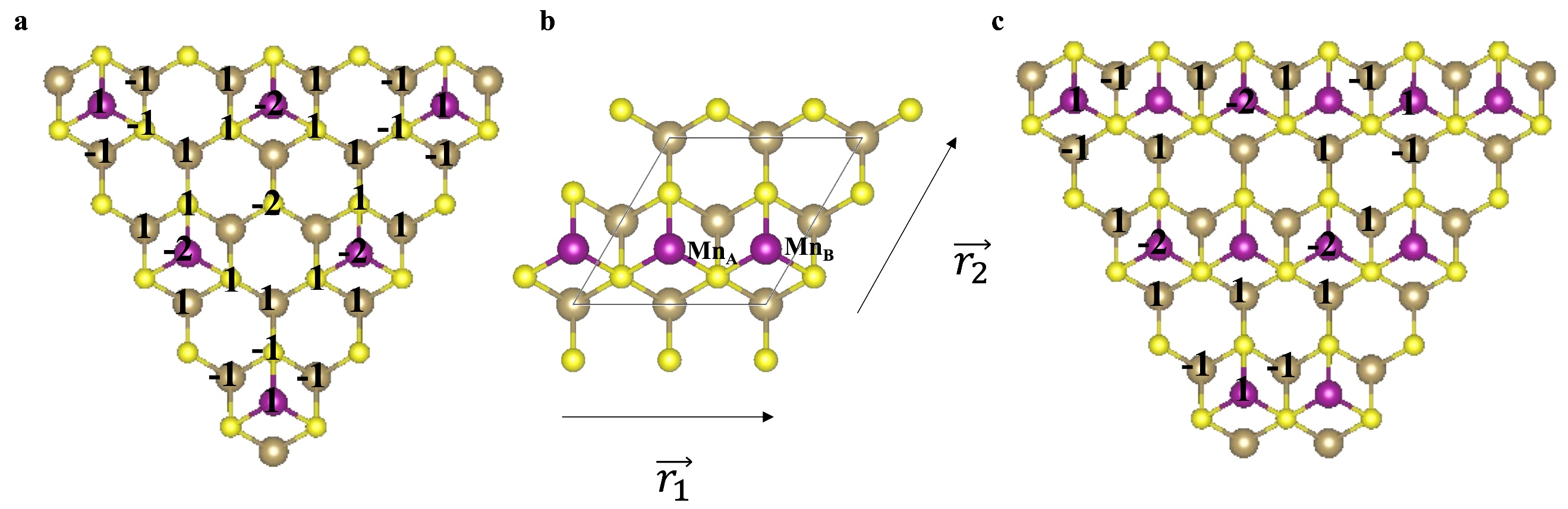


Fig. S10: Localization of flat bands in other intercalation cases. **a**, The localized state of interstitial intercalation (2H_c_-TMD) with amplitude/phase of each atom labeled, corresponding to $\varepsilon_{eff}=\varepsilon_{S}$ solution. **b**, Tight bind structure of the interstitial intercalation with multiple intercalants (labeled by Mn_A_ and Mn_B_) in the unit cell. **c**, The localized state (same as single intercalant case) of the structure shown in **b**.


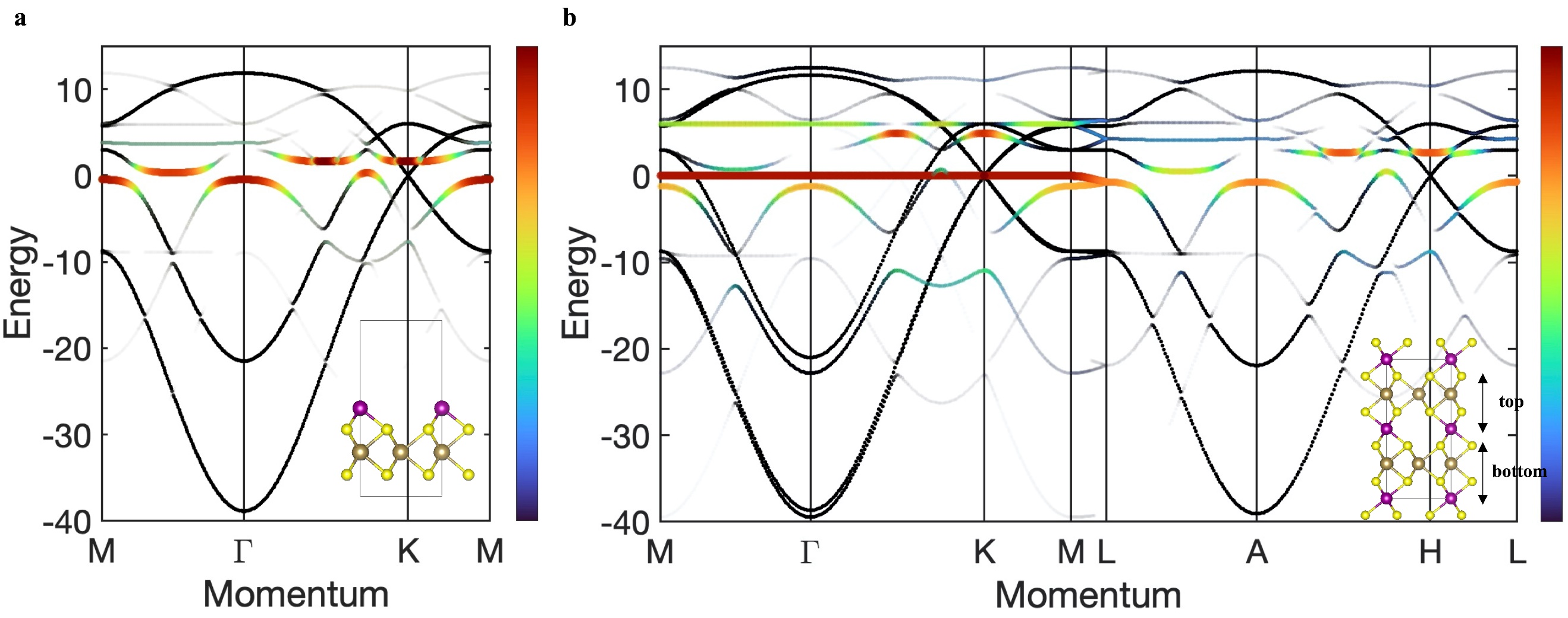


Fig. S11: Band structures of **a** monolayer and **b** bulk intercalated TMD. Insets are the structures used for tight binding modeling, and the bottom/top layer positions are indicated. Mn flat bands are marked by the yellowish color. The parameters used here are $onsite=(0, -6, 0)$ eV, $t=(-5, -3)$ eV and $t_{NNN}=(0, -3, 0)$ eV.


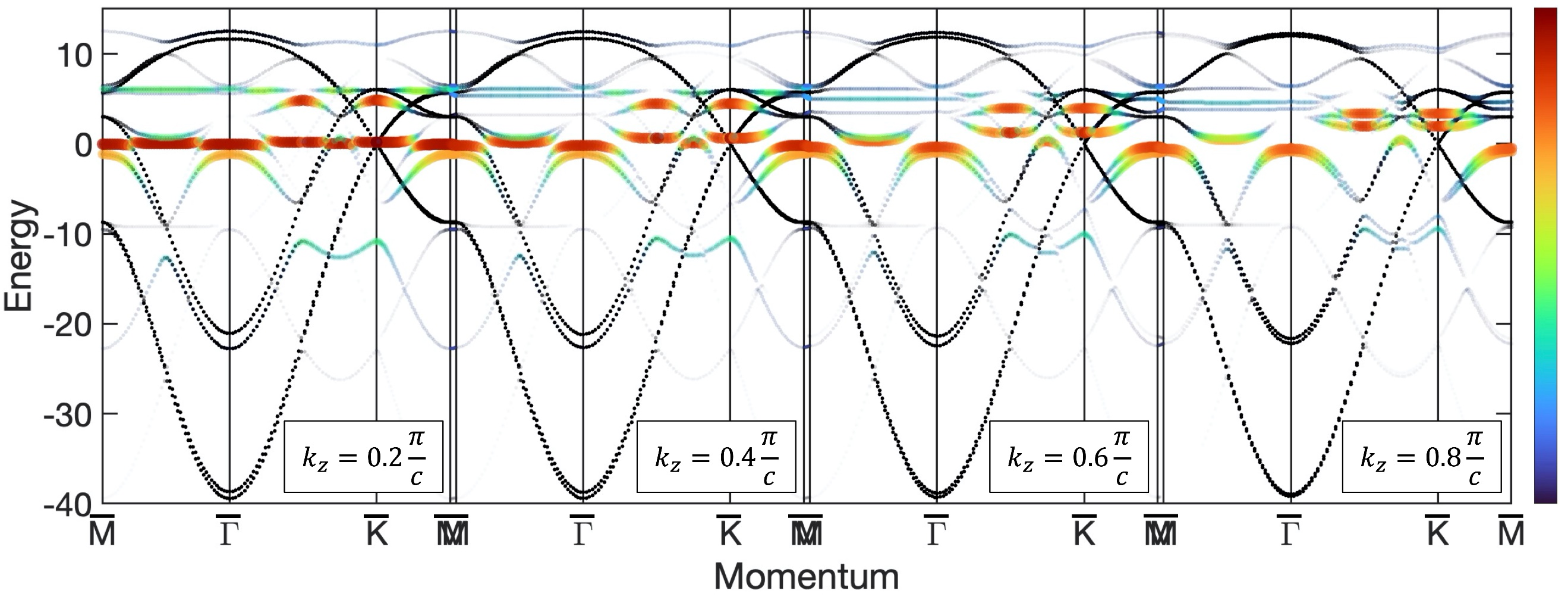
Fig. S12: Tight binding band structures of bulk intercalated TMD. The momentum k_z_ is given for each band structure. Mn flat bands are marked by the yellowish color. The parameters used here are $onsite=(0, -6, 0)$ eV, $t=(-5, -3)$ eV and $t_{NNN}=(0, -3, 0)$ eV.


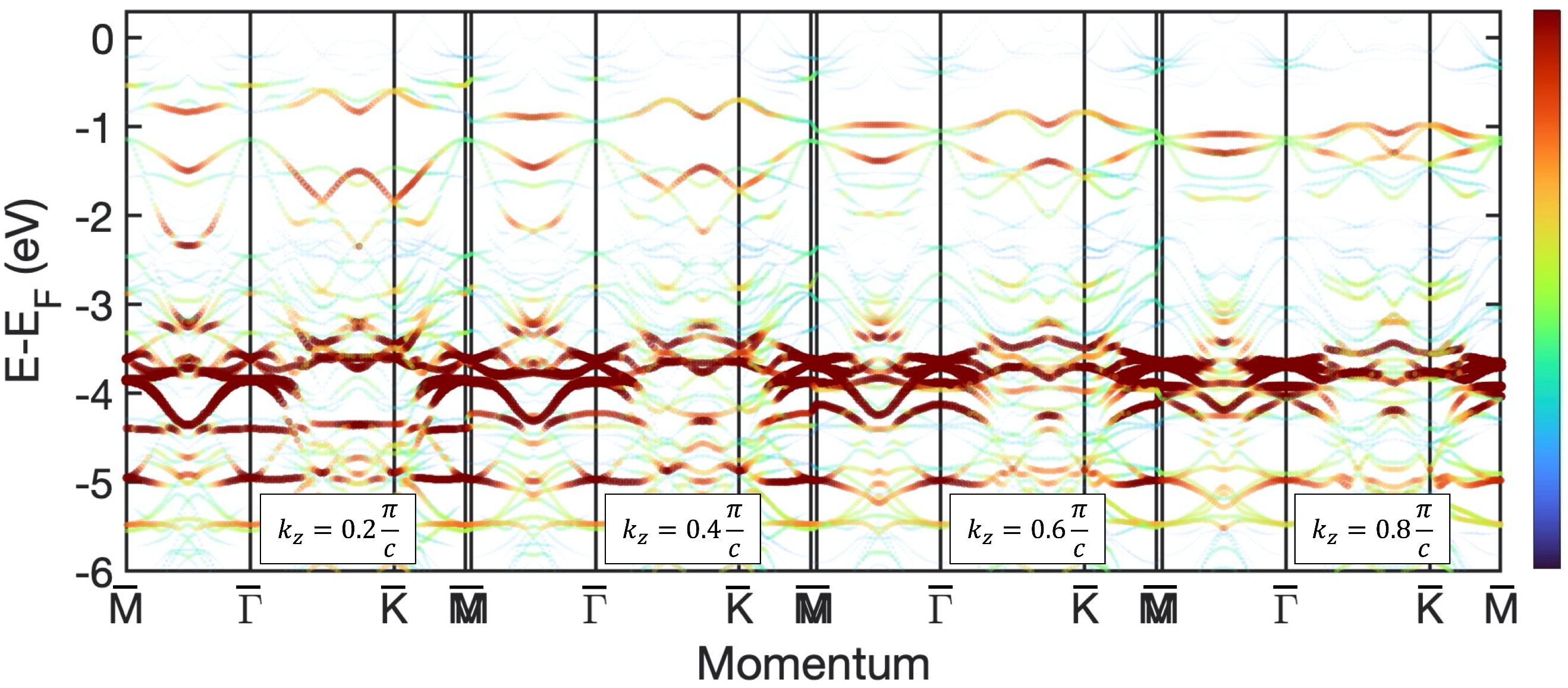


Fig. S13: DFT band structures of Mn_1/4_TaS_2_ at different k_z_. The band structure with spin down component below the Fermi level is given. The line width and color represent flat band weight projections of Mn.


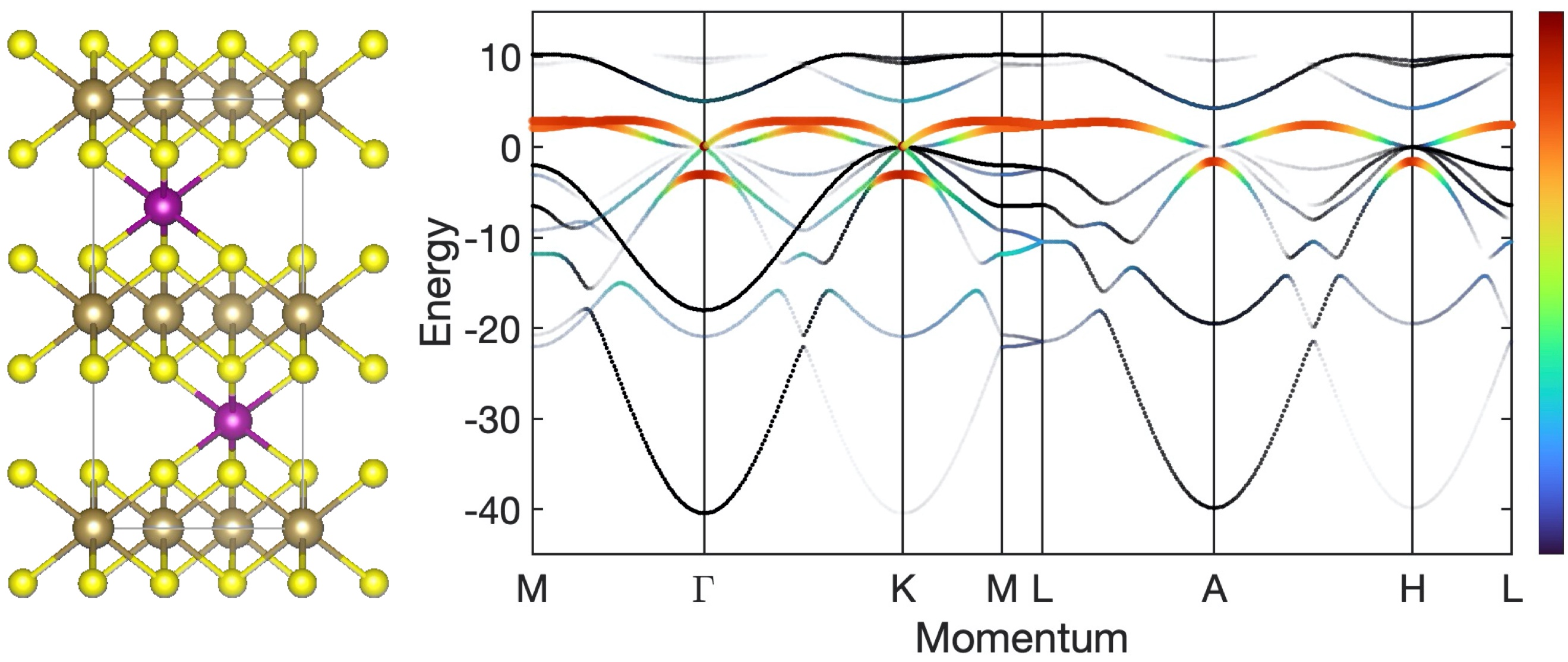


Fig. S14: Tight binding model and band structure of $\sqrt{3}\times\sqrt{3}$ T-TMD. The unit cell has two T phase layers due to intercalants misalignment. Line width and color represent Mn weight projection to the flat band. The parameters used here are $onsite=(0, -6, 0)$ eV, $t=(-5, -3)$ eV and $t_{NNN}=(-3, -2, -2)$ eV.

**Reference**

1. [Farjam, M. "Projection operator approach to unfolding supercell band structures." *arXiv preprint arXiv:1504.04937* (2015).](https://arxiv.org/abs/1504.04937)
2. [Rhim, Jun-Won, and Bohm-Jung Yang. "Classification of flat bands according to the band-crossing singularity of Bloch wave functions." *Physical Review B* 99.4 (2019): 045107.](https://journals.aps.org/prb/abstract/10.1103/PhysRevB.99.045107)
